# Maternal Cardiovascular Health During Pregnancy and Offspring Developmental Delay

**DOI:** 10.1001/jamanetworkopen.2026.18804

**Published:** 2026-06-23

**Authors:** Hisashi Ohseto, Mami Ishikuro, Geng Chen, Ippei Takahashi, Genki Shinoda, Aoi Noda, Keiko Murakami, Masatsugu Orui, Masato Takase, Noriyuki Iwama, Masahiro Kikuya, Hirohito Metoki, Atsushi Hozawa, Taku Obara, Shinichi Kuriyama

**Affiliations:** 1 Division of Disaster Public Health, International Research Institute of Disaster Science, Tohoku University, Sendai, Miyagi, Japan; 2 Division of Molecular Epidemiology, Environment and Genome Research Center, Graduate School of Medicine, Tohoku University, Sendai, Miyagi, Japan; 3 Department of Preventive Medicine and Epidemiology, Tohoku Medical Megabank Organization, Tohoku University, Sendai, Miyagi, Japan; 4 Department of Pharmaceutical Sciences, Tohoku University Hospital, Sendai, Miyagi, Japan; 5 Department of Health and Social Behavior, School of Public Health, Graduate School of Medicine, The University of Tokyo, Bunkyo-ku, Tokyo, Japan; 6 Center for Maternal and Perinatal Medicine, Tohoku University Hospital, Sendai, Miyagi, Japan; 7 Department of Hygiene and Public Health, Teikyo University School of Medicine, Itabashi-ku, Tokyo, Japan; 8 Division of Public Health, Hygiene and Epidemiology, Faculty of Medicine, Tohoku Medical and Pharmaceutical University, Sendai, Miyagi, Japan; 9 Division of Epidemiology, School of Public Health, Graduate School of Medicine, Tohoku University, Sendai, Miyagi, Japan

## Abstract

**Importance:**

Developmental delay affects daily living, social functioning, and mental health. Maternal cardiovascular health (CVH) during pregnancy may indicate an adverse intrauterine environment, but its association with developmental delay is unclear.

**Objective:**

To examine the association between maternal CVH during pregnancy and developmental delay in offspring at 4 years of age.

**Design, Setting, and Participants:**

This cohort study enrolled patients between July 19, 2013, and March 31, 2017, with 5 years of follow-up, at obstetric hospitals and clinics in Miyagi Prefecture, Japan. Participants included eligible mother and offspring pairs enrolled in the Tohoku Medical Megabank Project Birth and Three-Generation Cohort Study. Offspring were followed up until age 4 years. Data analyses were conducted from November 12, 2024, to March 24, 2026.

**Exposure:**

Maternal CVH during pregnancy, which was assessed using Life’s Essential 8 metrics (diet, physical activity, nicotine exposure, sleep health, body mass index, blood lipids, blood glucose, and blood pressure). Each metric was scored on a scale of 0 (least favorable) to 100 (most favorable), and these scores were used to categorize mothers as having high (80-100), moderate (50-79), or low (0-49) CVH.

**Main Outcomes and Measures:**

Developmental delay at age 4 years, which was evaluated by the mother using the validated Japanese version of Ages and Stages Questionnaire, Third Edition. This instrument has 5 domains: communication, gross motor, fine motor, problem solving, and personal-social skills. Domain-specific delay was defined as 2 or more SDs below the mean score, and developmental delay in total was defined as delay in 1 or more of the 5 domains.

**Results:**

Among 19 160 eligible mother and offspring pairs, 8238 (43.0%) were analyzed. Offspring were assessed at a mean (SD) age of 4.1 (0.2) years and included 4299 males (52.2%). Among mothers with high, moderate, and low CVH, 154 (8.8%), 763 (12.1%), and 33 (16.8%), respectively, had offspring with developmental delay in total. Compared with high CVH, moderate (risk ratio [RR], 1.30; 95% CI, 1.09-1.54) and low (RR, 1.62; 95% CI, 1.11-2.36) CVH during pregnancy were associated with developmental delay in total. Low CVH was associated with higher prevalence of developmental delay across all 5 domains, with personal-social domain having the largest effect size (RR, 2.23; 95% CI, 1.23-4.07; *P* for trend = .002) and communication domain having the smallest effect size (RR, 1.40; 95% CI, 0.69-2.85; *P* for trend = .03).

**Conclusions and Relevance:**

In this cohort study of mother and offspring pairs in Japan, better maternal CVH during pregnancy was associated with a lower risk of offspring developmental delay at age 4 years.

## Key Points

**Question:**

Is maternal cardiovascular health (CVH) during pregnancy associated with developmental delay in offspring at age 4 years?

**Findings:**

In this cohort study of 8238 mother and offspring pairs, developmental delay occurred in offspring of 8.8% of mothers with high CVH, 12.1% with moderate CVH, and 16.8% with low CVH. Compared with high CVH, moderate and low CVH during pregnancy were associated with developmental delay in total.

**Meaning:**

The findings showed that poor maternal CVH during pregnancy was associated with a higher risk of developmental delay in early childhood.

## Teaser

This cohort study assesses whether maternal cardiovascular health during pregnancy is associated with a delay in communication, gross motor, fine motor, problem solving, and personal-social skills among offspring in participants of the Tohoku Medical Megabank Project Birth and Three-Generation Cohort Study in Japan.

## Introduction

Developmental delay is a common condition in which development is delayed in 1 or more areas of motor skills, mental abilities, or language compared with skills of peers of the same age group. It affects approximately 15% of US youths,^1,2,3^ and the prevalence is increasing.^4,5^ It also contributes to reduced peer interactions,^6^ educational challenges,^6,7,8^ socioeconomic disadvantages,^9^ and psychiatric disorders in children and adolescents,^10,11^ posing a substantial public health concern. Thus, identification of modifiable risk factors and development of strategies for appropriate intervention and treatment are crucial. Identified risk factors include genetic^12,13^ and sociodemographic factors,^14,15^ perinatal disorders,^16^ and an adverse intrauterine environment.^17,18,19^ In particular, as highlighted by the developmental origins hypothesis,^20^ the intrauterine environment may affect the long-term health outcomes of offspring as well as neural and behavioral development,^21^ underscoring its role as an essential early intervention point in clinical practice.

Cardiovascular health (CVH) has attracted attention as an indicator of maternal health during the perinatal period.^22,23,24^ In 2013, the American Heart Association proposed Life’s Simple 7 (LS7) as a CVH metric that assesses the following easily obtainable clinical variables collectively: diet, physical activity, nicotine exposure, body mass index (BMI), blood lipids, blood glucose, and blood pressure (BP).^25^ When optimal, these variables are associated with greater cardiovascular disease–free survival,^26,27^ greater total longevity,^26,27^ and higher quality of life,^28^ affecting an individual’s lifespan from childhood to adulthood and therefore potentially serving as targets for clinical intervention. The usefulness of LS7 in the perinatal period has also been established, showing associations with adverse pregnancy outcomes and CVH in offspring.^23,24^ In 2022, the American Heart Association introduced an updated version of LS7—Life’s Essential 8 (LE8)—by adding the component of sleep health.^25^ Previous research has demonstrated an association between LE8 and adverse pregnancy outcomes.^22^ This finding suggests that maternal CVH during pregnancy may not only reflect maternal health but also provide a comprehensive assessment of the intrauterine environment, making it an important indicator of the long-term health and development of offspring. However, to date, no studies have demonstrated an association between maternal CVH during pregnancy and developmental delay in offspring. Therefore, the aim of the present study was to examine the association between maternal CVH during pregnancy, as assessed via LE8, and developmental delay in offspring at 4 years of age.

## Methods

### Participants

Between July 19, 2013, and March 31, 2017, pregnant women residing in Miyagi Prefecture, Japan, were enrolled in the Tohoku Medical Megabank Project Birth and Three-Generation (TMM BirThree) Cohort Study, a single, population-based prospective cohort.^29,30^ The Tohoku Medical Megabank Organization Ethics Committee approved this cohort study. All participants provided written informed consent. This study was conducted in accordance with the Declaration of Helsinki.^31^ We followed the Strengthening the Reporting of Observational Studies in Epidemiology (STROBE) reporting guideline.

Recruitment was conducted through approximately 50 collaborating obstetric hospitals and clinics. Women were excluded if they withdrew consent; had incomplete data on delivery outcomes or blood sampling; experienced a multiple birth, miscarriage, or stillbirth; or if their offspring presented with confirmed congenital anomalies at birth or at 1 month of age. We defined 32 weeks’ gestation as a landmark time point and restricted the analysis to pregnancies ongoing at that time, excluding deliveries occurring at or before 32 weeks’ gestation and those with blood samples collected after 32 weeks’ gestation. This landmark approach was adopted to define maternal CVH using information collected prior to the landmark time point and to reduce the association between late-pregnancy complications and CVH components, consistent with previous research.^23,24^ We further excluded individuals with missing information on CVH indicators or offspring developmental outcomes.

### CVH Definitions

CVH was evaluated using the LE8 framework, which includes 8 metrics: diet, physical activity, nicotine exposure, sleep health, BMI, blood lipids, blood glucose, and BP (eTable 1 in Supplement 1).^25^ CVH was assessed during pregnancy following the approach used in a previous study.^22^ Self-reported questionnaires assessed diet, physical activity, nicotine exposure, and sleep health during pregnancy. Dietary intake was assessed using the 8-item Japanese Diet Index, a validated measure of adherence to the Japanese dietary pattern that has been associated with reduced risk of cardiovascular disease–related mortality.^32,33,34^ Prepregnancy BMI and BP measured before 20 weeks’ gestation were extracted from antenatal medical records. Blood samples collected during pregnancy as part of the TMM BirThree Cohort Study were used to determine lipids and glucose levels.^29^ For secondary analyses, postpartum CVH was assessed using self-reported questionnaires and physiological and biological measurements conducted at the TMM BirThree Cohort Study assessment center during the postpartum period. Each LE8 element was scored on a scale of 0 (least favorable) to 100 (most favorable), and the overall CVH score was calculated as the arithmetic mean of the 8 metrics of the LE8.^25^ Participants were categorized into 3 CVH groups based on this overall score: high (80-100), moderate (50-79), and low (0-49).^25^

### Outcomes

The primary outcome was developmental delay when the offspring reached 4 years of age, as assessed by the mother using the validated Japanese version of the Ages and Stages Questionnaire, Third Edition (ASQ-3).^35^ The ASQ-3 screens for developmental delay in children aged 1 to 66 months and covers 5 key domains: communication, gross motor, fine motor, problem solving, and personal-social skills.^36^ Each domain comprises 6 questions, scored as 10 (yes), 5 (sometimes), or 0 (not yet) points, yielding a total score ranging from 0 (none of the assessed skills was achieved) to 60 (all of the assessed skills were achieved). If 1 or 2 items were unanswered, the domain score was adjusted by multiplying the sum of the available scores by 1.2 or 1.5.^36^ Developmental delay in a domain was defined as a score at least 2 SDs below the mean, to be in line with the established screening criteria of the ASQ-3 and to ensure clinical interpretability.^36^ Offspring with delay in 1 or more domains were classified as having developmental delay in total.

### Covariates

Covariates included maternal age at conception (<35 or ≥35 years), educational level (≤high school diploma, junior or vocational college degree, or ≥university degree), annual household income (>4 million or ≤4 million yen [approximately US $35 600 in 2017]), alcohol consumption during pregnancy (yes or no), psychological distress during pregnancy (yes or no), social isolation during pregnancy (yes or no), parity (primipara or multiparous), and conception via in vitro fertilization or intracytoplasmic sperm injection (IVF or ICSI; yes or no) as well as offspring sex (male or female) and offspring’s maternal and paternal family history of developmental disorders. Maternal age at conception, maternal parity, and offspring sex were extracted from medical records, whereas data for other variables were obtained through self-administered questionnaires during pregnancy. Maternal psychological distress was evaluated using the 6-item Kessler Psychological Distress Scale,^37,38^ scored on a 5-point Likert scale, yielding a total score between 0 and 24, with scores of 5 or higher indicating the presence of psychological distress. Social isolation was measured with the 6-item Lubben Social Network Scale,^39^ which uses a 6-point Likert response format with a total score of 0 to 30, with values of 11 or lower indicating social isolation.

### Statistical Analysis

CVH scores during pregnancy and post partum, covariates, and prevalence of developmental delay were compared across CVH levels during pregnancy. Pearson correlation coefficients (*r*) were calculated between each component CVH score during pregnancy and post partum. To assess potential nonlinear associations between CVH scores during pregnancy and developmental delay prevalence, we applied natural cubic splines using the ns function of the splines package in R (R Project for Statistical Computing). Knots were manually placed at values of 50 and 80, according to the definition of CVH levels.

Missing data were handled using multiple imputation by chained equations implemented in the mice package in R.^40^ Twenty imputed datasets were generated, with 50 iterations per dataset. The imputation model included the exposure (pregnancy CVH score), the outcome (offspring developmental delay at age 4 years), and all covariates as factors. Imputation models were specified according to variable type (predictive mean matching for continuous variables; logistic, multinomial, or ordinal regression for categorical variables), assuming data were missing at random, conditional on variables included in the imputation model.

To account for confounding, inverse probability of treatment weighting (IPTW) analysis was applied to estimate marginal associations. Propensity scores were estimated using multinomial logistic regression including the covariates. Stabilized inverse probability of treatment weights were calculated as the ratio of the marginal probability of the observed exposure to the conditional probability of exposure given covariates. To reduce the impact of extreme weights, weights were truncated at the first and 99th percentiles. Weighted Poisson regression models with robust variance were then fitted to estimate marginal risk ratios (RRs).

Sensitivity analyses were conducted to assess the robustness of the primary findings. First, covariate-adjusted Poisson regression models with robust variance were fitted without weighting to estimate conditional associations. These models included the same set of covariates as in the primary IPTW model. Second, to assess potential selection bias from exclusions related to missing data, we conducted a sensitivity analysis using inverse probability of selection weighting, with inclusion probabilities estimated using the same covariates as in the primary IPTW model. Third, additional landmark analyses were performed using alternative gestational ages of 20, 28, and 34 weeks. Landmarks at 28 and 34 weeks’ gestation were selected to be adjacent to the primary landmark of 32-weeks’ gestation, whereas the landmark of 20 weeks’ gestation represents an earlier gestational stage before many perinatal complications. Fourth, analyses were repeated after restricting the sample to term births only and by additionally adjusting for gestational age at delivery to evaluate whether the associations were attributable to preterm birth or gestational duration. Fifth, to examine whether the observed associations were due to any single CVH component, leave-one-component-out analyses were conducted by recalculating the CVH score using 7 of the 8 LE8 metrics. Moreover, a separate sensitivity analysis was conducted using the DASH (Dietary Approaches to Stop Hypertension) score as an alternative dietary component.

As a secondary analysis, we examined individual CVH components to explore whether specific domains might disproportionately affect the association between overall CVH and offspring developmental delay. Associations were estimated using unweighted covariate-adjusted Poisson regression models with robust variance. Moreover, we conducted a joint exposure analysis examining CVH levels during pregnancy and post partum simultaneously. CVH at each time point was dichotomized into high vs moderate or low, yielding 4 joint exposure groups.

A 2-sided *P* < .05 was considered to be statistically significant. All analyses were performed from November 12, 2024, to March 24, 2026, using R, version 4.1.2 (R Project for Statistical Computing).

## Results

**Figure 1.**
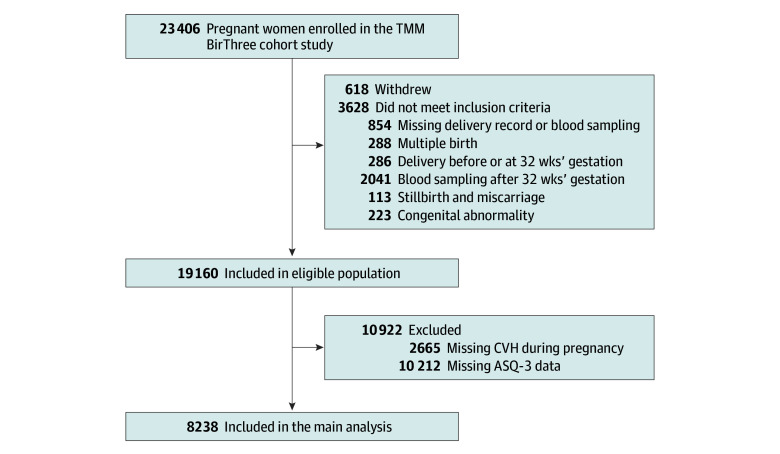
Flowchart of the Cohort Eligible for Main Analysis ASQ-3 indicates Ages and Stages Questionnaire, Third Edition; CVH, cardiovascular health; TMM BirThree, Tohoku Medical Megabank Project Birth and Three-Generation Cohort Study.

Among the 23 406 mother and offspring pairs enrolled in the TMM BirThree Cohort Study, 19 160 were eligible after applying exclusion criteria. Of these pairs, 8238 (43.0%) were included in the final analysis. The remaining 10 922 participants (57.0%) were excluded from the eligible population due to missing data on CVH and the ASQ-3 outcome, with some overlap between these groups (Figure 1). Offspring were assessed at a mean (SD) age of 4.1 (0.2) years and included 3939 females (47.8%) and 4299 males (52.2%) (eTable 2 in Supplement 1). The mean (SD) gestational age was 27.0 (4.4) weeks at questionnaire assessment, 19.4 (4.9) weeks at the laboratory assessment, and 11.3 (2.1) weeks at the BP measurement. Among the 8238 mothers, 1752 (21.3%) had high CVH, 6290 (76.4%) had moderate CVH, and 196 (2.4%) had low CVH (Table 1; eFigure 1 in Supplement 1). Mothers with low CVH during pregnancy compared with those in the moderate or high CVH groups were older at conception, had lower educational level and annual household income, experienced higher psychological distress, were more socially isolated, were primiparous, and were more likely to have conceived via IVF or ICSI. Among participants with high, moderate, and low CVH, 154 (8.8%), 763 (12.1%), and 33 (16.8%), respectively, produced offspring with developmental delay in total (Table 1).

**Table 1.** Maternal and Offspring Characteristics by Maternal Cardiovascular Health Levels During Pregnancy in the Unweighted and Inverse Probability of Treatment Weighted Samples

Characteristic	Unweighted sample, No. (%)				Inverse probability of treatment weighted sample, No. (%)				Missing ratio
	High CVH during pregnancy (n = 1752)^a^	Moderate CVH during pregnancy (n = 6290)^a^	Low CVH during pregnancy (n = 196)^a^	SMD	High CVH during pregnancy (n = 1729.9)^a^	Moderate CVH during pregnancy (n = 6290.1)^a^	Low CVH during pregnancy (n = 192.5)^a^	SMD	
**Maternal**									
Advanced age at conception	439 (25.1)	1803 (28.7)	65 (33.2)	0.119	475.9 (27.5)	1761.7 (28.0)	55.9 (29.0)	0.023	0.0
Educational level				0.431				0.155	11.3
≤High school diploma	382 (24.4)	1758 (31.5)	83 (50.3)		520.2 (30.1)	1918.6 (30.5)	74.9 (38.9)		
Junior or vocational college degree	644 (41.2)	2200 (39.4)	61 (37.0)		682.7 (39.5)	2491.7 (39.6)	76.1 (39.5)		
≥University degree	537 (34.4)	1623 (29.1)	21 (12.7)		527.0 (30.5)	1879.8 (29.9)	41.5 (21.6)		
Annual household income <4 million yen^b^	485 (28.9)	2125 (35.2)	100 (52.4)	0.326	590.1 (34.1)	2161.8 (34.4)	79.2 (41.1)	0.097	3.9
Alcohol consumption during pregnancy	345 (19.7)	1286 (20.5)	30 (15.4)	0.089	356.6 (20.6)	1271.6 (20.2)	33.7 (17.5)	0.053	0.1
Psychological distress during pregnancy	407 (23.4)	1716 (27.4)	73 (37.4)	0.206	458.1 (26.5)	1687.7 (26.8)	62.1 (32.2)	0.084	0.6
Social isolation during pregnancy	285 (16.4)	1253 (20.1)	63 (32.5)	0.254	328.8 (19.0)	1230.3 (19.6)	45.8 (23.8)	0.078	0.7
Primipara	535 (30.5)	2553 (40.6)	80 (40.8)	0.144	647.6 (37.4)	2417.5 (38.4)	79.1 (41.1)	0.050	0.0
Conception via IVF or ICSI	62 (3.5)	368 (5.9)	21 (10.7)	0.189	89.3 (5.2)	344.8 (5.5)	13.8 (7.2)	0.055	0.0
**Offspring**									
Maternal and paternal family history of developmental disorders	2 (0.1)	11 (0.2)	0 (0.0)	0.043	3.6 (0.2)	17.5 (0.3)	0.6 (0.3)	0.015	10.6
Sex									
Female	824 (47.0)	3020 (48.0)	95 (48.5)	0.019	827.3 (47.8)	3008.3 (47.8)	92.8 (48.2)	0.005	0.0
Male	928 (53.0)	3270 (52.0)	101 (51.5)		902.6 (52.2)	3281.8 (52.2)	99.7 (51.8)		
Birth weight, mean (SD), g	3014.6 (386.0)	3034.4 (395.6)	3098.2 (425.8)	0.137	3008.1 (381.6)	3036.3 (395.6)	3124.7 (430.1)	0.191	0.0
Low birth weight	125 (7.1)	494 (7.9)	12 (6.1)	0.045	125.9 (7.3)	490.2 (7.8)	11.5 (6.0)	0.048	0.0
Gestational age, mean (SD), wk	39.2 (1.3)	39.2 (1.4)	39.1 (1.6)	0.078	39.2 (1.3)	39.2 (1.4)	39.1 (1.6)	0.073	0.0
Preterm birth	72 (4.1)	300 (4.8)	15 (7.7)	0.101	73.0 (4.2)	298.7 (4.7)	15.7 (8.2)	0.110	0.0
Postpartum CVH score, mean (SD), point	72.9 (8.1)	66.8 (9.9)	50.5 (12.2)	1.435	72.7 (8.1)	66.9 (9.9)	50.9 (12.3)	1.387	37.0
Developmental delay at age 4 y									
Total	154 (8.8)	763 (12.1)	33 (16.8)	0.162	161.2 (9.3)	754.7 (12.0)	29.3 (15.2)	0.121	0.0
Communication	48 (2.7)	262 (4.2)	10 (5.1)	0.082	50.4 (2.9)	258.5 (4.1)	8.5 (4.4)	0.053	0.0
Gross motor	47 (2.7)	269 (4.3)	12 (6.1)	0.113	52.1 (3.0)	266.2 (4.2)	10.5 (5.5)	0.082	0.0
Fine motor	58 (3.3)	328 (5.2)	15 (7.7)	0.129	62.5 (3.6)	323.1 (5.1)	12.3 (6.4)	0.085	0.0
Problem solving	42 (2.4)	250 (4.0)	14 (7.1)	0.151	46.7 (2.7)	247.1 (3.9)	11.0 (5.7)	0.100	0.0
Personal-social	52 (3.0)	293 (4.7)	15 (7.7)	0.141	53.8 (3.1)	288.1 (4.6)	13.1 (6.8)	0.115	0.0

Abbreviations: CVH, cardiovascular health; ICSI, intracytoplasmic sperm injection; IVF, in vitro fertilization; SMD, standardized mean differences.

^a^ CVH score during pregnancy was used to categorize participants into these groups: high (80-100), moderate (50-79), and low (0-49).

^b^ To convert yen to dollars, multiply by 0.0089 (based on 2017 average exchange rate).

**Figure 2.**
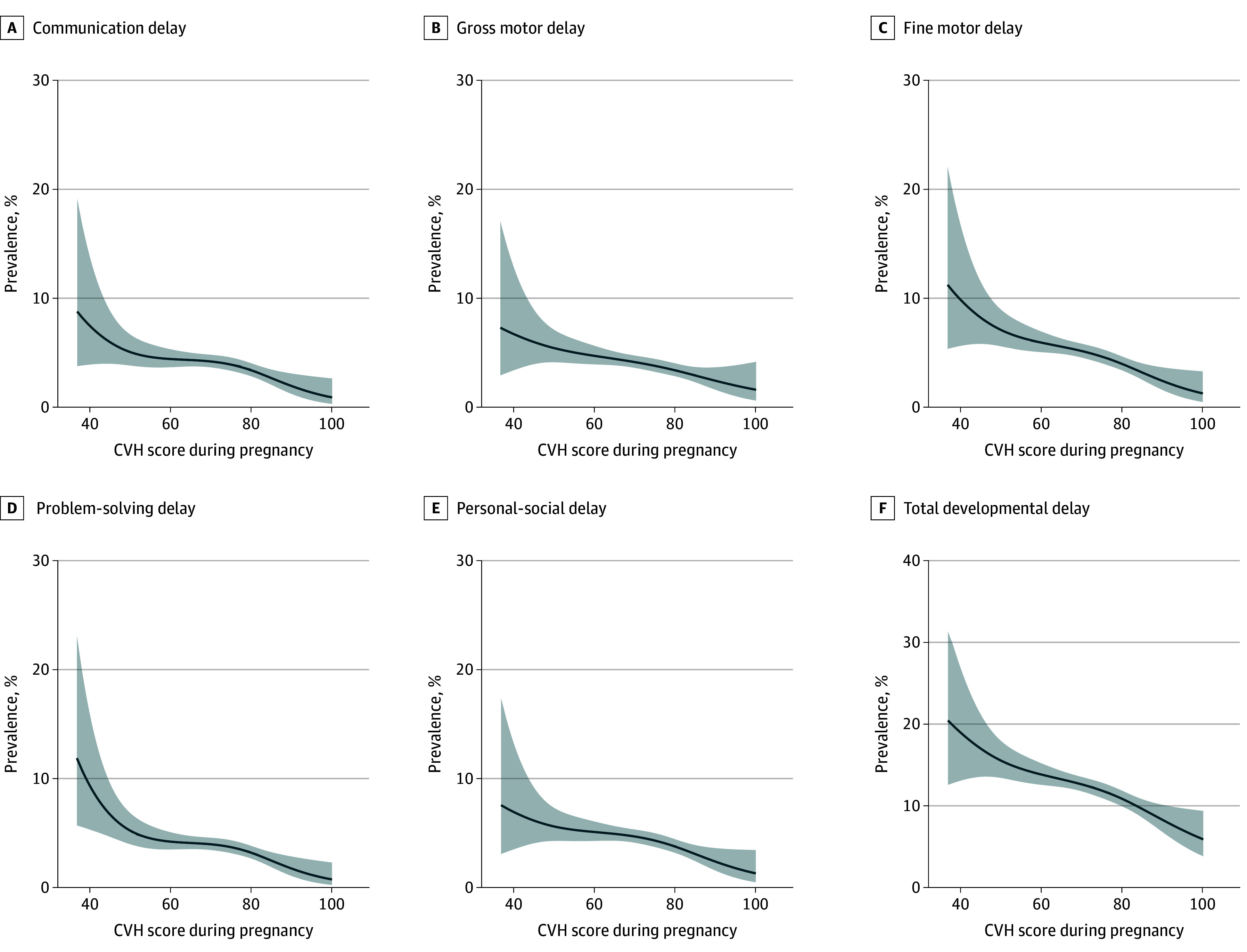
Cubic Splines Showing Nonlinear Association Between Cardiovascular Health (CVH) Score During Pregnancy and Developmental Delay Prevalence Natural cubic splines were applied, with knots manually placed at values of 50 and 80, according to CVH levels definition. Developmental delay was measured using 5 domains in the Ages and Stages Questionnaire, Third Edition. Delay in total was defined as any delay in 1 or more of the 5 domains. Shaded areas represent 95% CIs.

After IPTW, most variables included in the propensity score model achieved adequate balance (standardized mean difference [SMD] <0.100), whereas educational level showed a higher SMD of 0.155 (Table 1). The highest missingness ratios were observed for maternal educational level (11.3) and family history of developmental disorders (10.6) (Table 1). Postpartum CVH and ASQ-3 scores were assessed at a median (IQR) of 3.3 (2.7-4.1) years and 4.1 (4.0-4.2) years after delivery, respectively. When comparing components of the CVH score measured during pregnancy and post partum, all components showed significant correlations with each other (eg, Diet: *r* = 0.34, *P* < .001; BMI: *r* = 0.79, *P* < .001) (eFigure 2 in Supplement 1). According to the model using cubic splines, the association between CVH scores and developmental delay prevalence was uniformly decreasing. The slope was steeper below the value of 50, leveled off around 50 to 60, remained gradual, and then changed around 70 to 80, becoming steeper thereafter (Figure 2). The characteristics of the study population differed from those of the excluded participants due to missing exposure or outcome data, with the included participants tending to be older and to have higher educational level and annual income as well as lower levels of psychological distress, among other differences (eTables 2-4 in Supplement 1). Some variables showed different patterns, depending on whether the missing data involved exposure or outcome variables, suggesting possible differences in missingness mechanisms.

In the IPTW analysis (Table 2), moderate CVH (RR, 1.30; 95% CI, 1.09-1.54) and low CVH (RR, 1.62; 95% CI, 1.11-2.36) during pregnancy were associated with developmental delay in total (*P* for trend < .001). Low CVH was also associated with a higher prevalence of developmental delay across all 5 domains. Of these associations, the personal-social domain had the largest effect size (RR, 2.23; 95% CI, 1.23-4.07; *P* for trend = .002) while the communication domain had the smallest effect size (RR, 1.40; 95% CI, 0.69-2.85; *P* for trend = .03), when comparing low to high CVH.

**Table 2.** Association Between Maternal Cardiovascular Health Levels During Pregnancy and Offspring Developmental Delay at Age 4 Years

Developmental delay and CVH level during pregnancy^a^	Crude			Inverse probability of treatment weighted		
	Estimated RR (95% CI)	*P* value	*P* for trend	Estimated RR (95% CI)	*P* value	*P* for trend
Total						
High	1 [Reference]	NA	<.001	1 [Reference]	NA	<.001
Moderate	1.38 (1.17-1.63)	<.001		1.30 (1.09-1.54)	.003	
Low	1.92 (1.36-2.71)	<.001		1.62 (1.11-2.36)	.01	
Communication						
High	1 [Reference]	NA	.003	1 [Reference]	NA	.03
Moderate	1.52 (1.12-2.06)	.007		1.41 (1.03-1.94)	.03	
Low	1.86 (0.96-3.62)	.07		1.40 (0.69-2.85)	.35	
Gross motor						
High	1 [Reference]	NA	<.001	1 [Reference]	NA	.02
Moderate	1.59 (1.17-2.16)	.003		1.41 (1.03-1.94)	.03	
Low	2.28 (1.23-4.23)	.009		1.81 (0.93-3.52)	.08	
Fine motor						
High	1 [Reference]	NA	<.001	1 [Reference]	NA	.006
Moderate	1.58 (1.20-2.07)	.001		1.43 (1.08-1.90)	.01	
Low	2.31 (1.34-4.00)	.003		1.78 (1.00-3.18)	.05	
Problem solving						
High	1 [Reference]	NA	<.001	1 [Reference]	NA	.009
Moderate	1.66 (1.20-2.29)	.002		1.46 (1.05-2.05)	.03	
Low	2.98 (1.66-5.36)	<.001		2.03 (1.08-3.81)	.03	
Personal-social						
High	1 [Reference]	NA	<.001	1 [Reference]	NA	.002
Moderate	1.57 (1.17-2.10)	.002		1.49 (1.10-2.01)	.009	
Low	2.58 (1.48-4.49)	<.001		2.23 (1.23-4.07)	.009	

Abbreviations: CVH, cardiovascular health; NA, not applicable; RR, risk ratio.

^a^ Developmental delay measured using 5 domains in the Ages and Stages Questionnaire, Third Edition.

**Figure 3.**
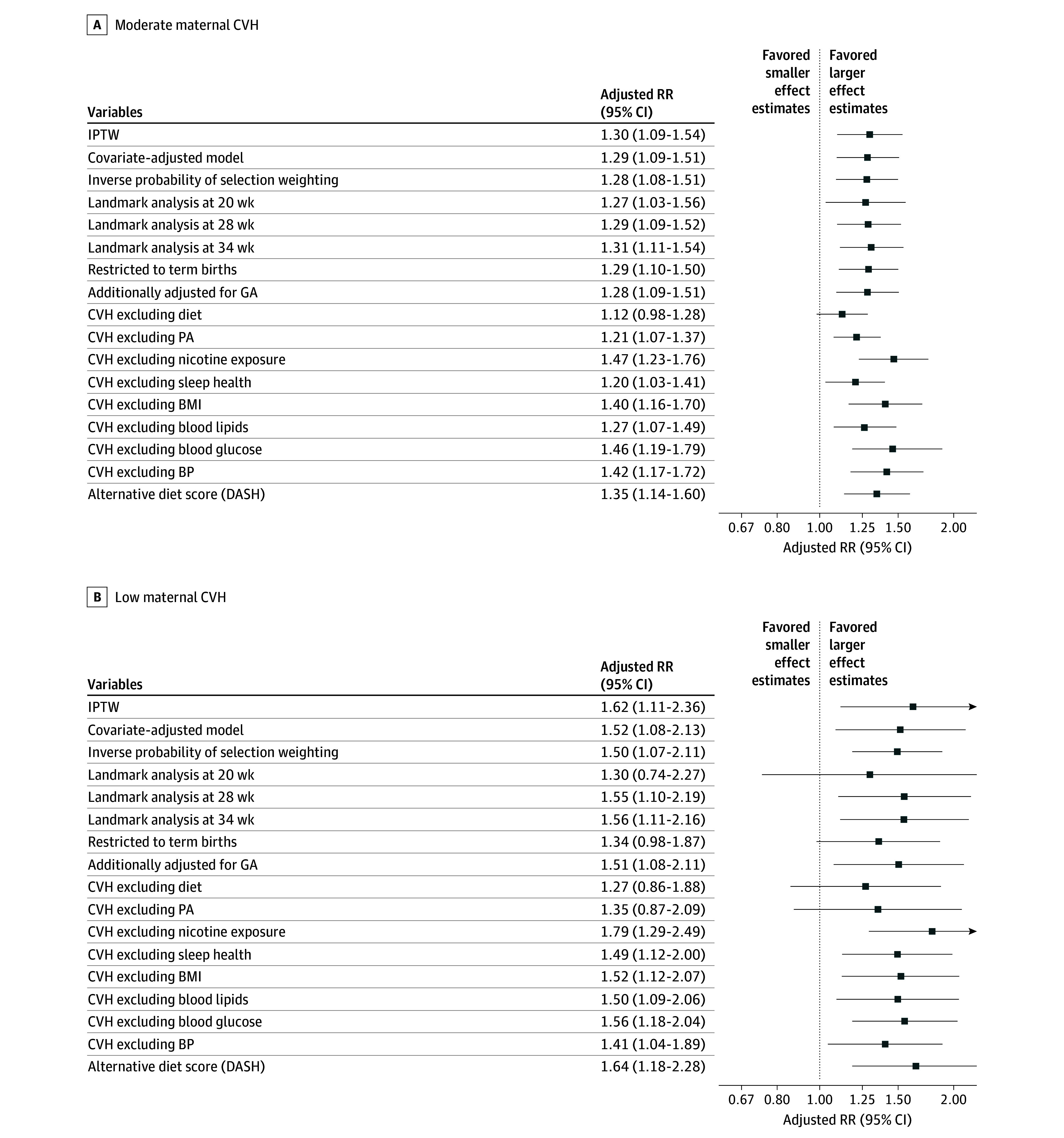
Dot Plots Showing Sensitivity Analyses of the Association Between Maternal Cardiovascular Health (CVH) and Offspring Developmental Delay in Total High CVH is the reference category. The point estimate of the inverse probability of treatment weighting (IPTW) model is indicated by a vertical dashed (reference) line within each CVH stratum. Circles represent adjusted risk ratios (RRs), and error bars represent 95% CIs. BMI indicates body mass index; BP, blood pressure; DASH, Dietary Approaches to Stop Hypertension; GA, gestational age; and PA, physical activity.

Sensitivity analyses yielded generally stable results (Figure 3). In the covariate-adjusted analyses, which targeted conditional associations, the direction and magnitude of the associations were largely consistent with those of the IPTW model, with RR estimates of 1.29 (95% CI, 1.09-1.51) for the moderate category and 1.52 (95% CI, 1.08-2.13) for the low category (eTable 5 in Supplement 1). Results were also unchanged when inverse probability of selection weighting was applied, with RR estimates of 1.28 (95% CI, 1.08-1.51) for the moderate category and 1.50 (95% CI, 1.07-2.11) for the low category (eTable 6 in Supplement 1). Across alternative landmark analyses, results were generally consistent, although effect sizes were slightly attenuated when the landmark was set at 20 weeks’ gestation (eTable 7 in Supplement 1). In analyses restricted to term births, the adjusted RR for the communication domain was null, whereas effect sizes for the other domains showed slight attenuation compared with the main analysis (eTable 8 in Supplement 1); additional adjustment for gestational age at delivery resulted in only modest attenuation of the estimates (eTable 9 in Supplement 1). In leave-one-component-out analyses, associations remained directionally consistent, suggesting that no single component played a dominant role in the findings, although effect sizes varied more than in other sensitivity analyses, indicating sensitivity to how the composite CVH score was defined (eTable 10 in Supplement 1). When the dietary component was alternatively defined using the DASH score, effect sizes were slightly larger; however, the overall conclusions remained unchanged (eTable 11 in Supplement 1).

In component-level analyses of associations with developmental delay, no individual CVH component had a large effect size, with RRs ranging from approximately 1.0 to 1.2 (eTable 12 in Supplement 1). Nicotine exposure showed an inverse association with developmental delay; however, these findings should be interpreted cautiously because the analyses were exploratory and not intended for component-specific causal association. In joint exposure analyses, associations for developmental delay in total were higher when CVH was suboptimal in both pregnancy and postpartum periods than in either period alone, although 95% CIs were wide (eTable 13 in Supplement 1). Across domains, RRs for the postpartum-only suboptimal group were not consistently elevated; when point estimates were increased, they were generally lower than those for the pregnancy-only group or suboptimal group for both periods (eTable 13 in Supplement 1).

## Discussion

This cohort study demonstrated that maternal CVH during pregnancy was associated with developmental delay in offspring at 4 years of age, with the personal-social domain having the largest effect size and the communication domain having the smallest effect size. Across multiple sensitivity analyses, the direction and magnitude of the associations were generally consistent with the primary findings. Component-level analyses did not suggest a single dominant factor, supporting the relevance of overall CVH as a composite indicator. In joint exposure analyses, effect sizes tended to be higher when CVH was suboptimal in both periods, although 95% CIs were wide.

Maternal CVH during pregnancy has been associated with perinatal diseases,^22,24^ highlighting the need for further investigations of its potential associations with offspring outcomes. The only previous study examining offspring outcomes found that maternal CVH during pregnancy was associated with offspring CVH at ages 10 to 14 years.^23^ However, that study lacked data for the period spanning birth to time of assessment, making it challenging to elucidate the underlying mechanisms. Our study offers new insights that suggest a potential pathway in which poor maternal CVH contributes to developmental delay in offspring, which may, in turn, contribute to deterioration of offspring CVH through factors such as preference for unhealthy diets.^41^ Given the established associations between developmental delay and poor academic performance^7,8^ and psychiatric disorders,^10,11^ maternal CVH during pregnancy has implications not only for the offspring CVH but also for psychological and psychiatric outcomes. This wide-ranging impact is consistent with the developmental origins hypothesis,^21^ underscoring the critical role of prenatal environments in shaping long-term health outcomes.

Maternal CVH during pregnancy was associated with all 5 ASQ-3 domains, indicating its wide-ranging role in offspring development. Fetal brain development and the localization of neural functions are gradually being clarified.^42,43^ In adults, problem-solving abilities are primarily localized in the prefrontal cortex,^44^ which begins forming as early as the first trimester of pregnancy.^45^ In contrast, the language network develops predominantly from the second trimester onward.^46^ This information suggests that the critical window for problem solving may occur earlier and therefore may be more biologically susceptible to the maternal environment compared with language development, potentially explaining the domain-specific differences observed. However, as knowledge of functional brain localization and fetal neurodevelopment continues to evolve, further research is warranted.

The joint exposure analyses suggest that suboptimal CVH during pregnancy may be more relevant to offspring development than suboptimal CVH confined to the postpartum period, although patterns differed across ASQ-3 domains. Some domains may be altered more by biological factors in utero, such as nutrient supply,^47^ inflammatory responses,^48^ and epigenetic modifications,^49^ while other domains may be shaped more by postnatal environmental factors, including parent-child interactions^50^ and learning support.^51^ Additionally, certain domains may exhibit greater plasticity in response to the postnatal environment.^52,53^ Therefore, prevention and intervention strategies should be tailored to the sensitivity and plasticity of each domain.

### Clinical Implications

The findings of this study align with previous research showing that individual components of CVH, such as diet,^54,55^ nicotine exposure,^56^ sleep,^57^ obesity,^58^ and hypertensive disorders of pregnancy,^16^ are associated with developmental delay in offspring. Maternal CVH during pregnancy not only integrates the combined outcome of these components but also serves as a practical clinical indicator for monitoring the effectiveness of outpatient guidance and treatment in complex clinical contexts.^25^ Previous studies have also demonstrated associations between maternal CVH and both perinatal diseases^22,24^ and offspring CVH,^23^ indicating that CVH should be actively used as a key protective measure of maternal and child health. Moreover, since developmental delay in areas such as gross motor and communication skills often improve with appropriate interventions,^59,60^ maternal CVH assessment can help identify offspring at risk of developmental delay, facilitating the implementation of treatment strategies for mothers and their offspring.

### Strengths and Limitations

This study has some strengths. To our knowledge, it is the first to demonstrate an association between maternal CVH during pregnancy and developmental delay in offspring. CVH was measured comprehensively using all LE8 metrics, and offspring development was measured using the ASQ-3, a validated and widely used measure that includes domain-specific evaluations, facilitating the exploration of physiological mechanisms in greater detail.

The study also has some limitations. First, selection bias is possible, as about half of eligible participants were excluded primarily because of incomplete outcome data. However, associations were similar in direction and magnitude across sensitivity analyses, which accounted for study inclusion and alternative definitions of the eligible population, suggesting that differential inclusion based on measured characteristics had limited implications for the results. Second, restricting the analysis to pregnancies ongoing at the landmark time may limit generalizability. Using pregnancy continuation as a condition for inclusion could introduce bias if unmeasured factors affect both reaching the landmark and offspring developmental outcomes.^61,62^ Furthermore, subclinical pregnancy complications may have begun before CVH assessment and could have changed the components of CVH, resulting in residual confounding by early disease processes. Third, offspring developmental delay was assessed using the ASQ-3, which is a screening instrument that identifies children at risk for developmental delay rather than providing a clinical diagnosis. Moreover, as the ASQ-3 is completed by the parent, responses may be affected by parental literacy, recall, and sociocultural perceptions of child development. Such factors may vary by educational level and socioeconomic status and could introduce differential measurement error. Developmental status at 4 years of age may change over time for some offspring, underscoring the importance of longer-term follow-up and, where feasible, complementary objective assessments.^63^

## Conclusions

In this cohort study of mother and offspring pairs in Japan, better maternal CVH during pregnancy was associated with a lower risk of offspring developmental delay at age 4 years. This association was observed across multiple developmental domains, suggesting that maternal CVH during pregnancy may be relevant to broad aspects of offspring development.

## Notes

Supplement 1. **eTable 1.** Cardiovascular Health Definition in This Study **eTable 2.** Characteristics of Included and Excluded Participants in the Eligible Population **eTable 3.** Characteristics of Participants With and Without Missing Exposure Data **eTable 4.** Characteristics of Participants With and Without Missing Outcome Data **eTable 5.** Conditional Associations Between Maternal Cardiovascular Health During Pregnancy and Offspring Developmental Delay at 4 Years **eTable 6.** Associations Between Maternal Cardiovascular Health During Pregnancy and Offspring Developmental Delay at 4 Years: Inverse Probability of Selection Weighting Analysis **eTable 7.** Associations Between Maternal Cardiovascular Health During Pregnancy and Offspring Developmental Delay at 4 Years: Alternative Gestational Age Landmark Cutoffs **eTable 8.** Associations Between Maternal Cardiovascular Health During Pregnancy and Offspring Developmental Delay at 4 Years Among Term Births **eTable 9.** Associations Between Maternal Cardiovascular Health During Pregnancy and Offspring Developmental Delay at 4 Years After Additional Adjustment for Gestational Age at Delivery **eTable 10.** Associations Between Maternal Cardiovascular Health During Pregnancy and Offspring Developmental Delay at 4 Years: Leave-One-Component-Out Analysis **eTable 11.** Associations Between Maternal Cardiovascular Health During Pregnancy and Offspring Developmental Delay at 4 Years Using the DASH Score for the Dietary Component **eTable 12.** Associations Between Individual Maternal Cardiovascular Health Components During Pregnancy and Offspring Developmental Delay at 4 Years **eTable 13.** Joint Associations of Maternal Cardiovascular Health During Pregnancy and Postpartum With Offspring Developmental Delay at 4 Years **eFigure 1.** Distribution of Maternal Cardiovascular Health Scores During Pregnancy **eFigure 2.** Heatmap of Correlations Between Cardiovascular Health Components During Pregnancy and Postpartum

Supplement 2.
Data Sharing Statement
