# Supplementary material for: Maternal Cardiovascular Health During Pregnancy and Offspring Developmental Delay
Source: JAMA Netw Open. 2026 Jun 23;9(6):e2618804. doi: 10.1001/jamanetworkopen.2026.18804 (PMC13291893; doi:10.1001/jamanetworkopen.2026.18804)
Supplement: Supplement 1. — eTable 1. Cardiovascular Health Definition in This Study eTable 2. Characteristics of Included and Excluded Participants in the Eligible Population eTable 3. Characteristics of Participants With and Without Missing Exposure Data eTable 4. Characteristics of Participants With and Without Missing Outcome Data eTable 5. Conditional Associations Between Maternal Cardiovascular Health During Pregnancy and Offspring Developmental Delay at 4 Years eTable 6. Associations Between Maternal Cardiovascular Health During Pregnancy and Offspring Developmental Delay at 4 Years: Inverse Probability of Selection Weighting Analysis eTable 7. Associations Between Maternal Cardiovascular Health During Pregnancy and Offspring Developmental Delay at 4 Years: Alternative Gestational Age Landmark Cutoffs eTable 8. Associations Between Maternal Cardiovascular Health During Pregnancy and Offspring Developmental Delay at 4 Years Among Term Births eTable 9. Associations Between Maternal Cardiovascular Health During Pregnancy and Offspring Developmental Delay at 4 Years After Additional Adjustment for Gestational Age at Delivery eTable 10. Associations Between Maternal Cardiovascular Health During Pregnancy and Offspring Developmental Delay at 4 Years: Leave-One-Component-Out Analysis eTable 11. Associations Between Maternal Cardiovascular Health During Pregnancy and Offspring Developmental Delay at 4 Years Using the DASH Score for the Dietary Component eTable 12. Associations Between Individual Maternal Cardiovascular Health Components During Pregnancy and Offspring Developmental Delay at 4 Years eTable 13. Joint Associations of Maternal Cardiovascular Health During Pregnancy and Postpartum With Offspring Developmental Delay at 4 Years eFigure 1. Distribution of Maternal Cardiovascular Health Scores During Pregnancy eFigure 2. Heatmap of Correlations Between Cardiovascular Health Components During Pregnancy and Postpartum [file jamanetwopen-e2618804-s001.pdf]

Supplemental Online Content

Ohseto H, Ishikuro M, Chen G, et al. Maternal cardiovascular health during pregnancy and offspring developmental delay. *JAMA Netw Open*. 2026;9(6):e2618804. doi:10.1001/jamanetworkopen.2026.18804

eTable 1. Cardiovascular Health Definition in This Study
eTable 2. Characteristics of Included and Excluded Participants in the Eligible Population
eTable 3. Characteristics of Participants With and Without Missing Exposure Data
eTable 4. Characteristics of Participants With and Without Missing Outcome Data
eTable 5. Conditional Associations Between Maternal Cardiovascular Health During Pregnancy and Offspring Developmental Delay at 4 Years
eTable 6. Associations Between Maternal Cardiovascular Health During Pregnancy and Offspring Developmental Delay at 4 Years: Inverse Probability of Selection Weighting Analysis
eTable 7. Associations Between Maternal Cardiovascular Health During Pregnancy and Offspring Developmental Delay at 4 Years: Alternative Gestational Age Landmark Cutoffs
eTable 8. Associations Between Maternal Cardiovascular Health During Pregnancy and Offspring Developmental Delay at 4 Years Among Term Births
eTable 9. Associations Between Maternal Cardiovascular Health During Pregnancy and Offspring Developmental Delay at 4 Years After Additional Adjustment for Gestational Age at Delivery
eTable 10. Associations Between Maternal Cardiovascular Health During Pregnancy and Offspring Developmental Delay at 4 Years: Leave-One-Component-Out Analysis
eTable 11. Associations Between Maternal Cardiovascular Health During Pregnancy and Offspring Developmental Delay at 4 Years Using the DASH Score for the Dietary Component
eTable 12. Associations Between Individual Maternal Cardiovascular Health Components During Pregnancy and Offspring Developmental Delay at 4 Years
eTable 13. Joint Associations of Maternal Cardiovascular Health During Pregnancy and Postpartum With Offspring Developmental Delay at 4 Years
eFigure 1. Distribution of Maternal Cardiovascular Health Scores During Pregnancy
eFigure 2. Heatmap of Correlations Between Cardiovascular Health Components During Pregnancy and Postpartum

This supplemental material has been provided by the authors to give readers additional information about their work.

© 2026 Ohseto H et al. *JAMA Network Open.*

eTable 1. Cardiovascular Health Definition in This Study

Components	Scoring systems
Diet	**Metrics:** 8-item Japanese Diet Index score<br><br>**Scoring:**<br><u>Points</u> <u>Quantile</u><br>100 ≥95th percentile (Most adherence to the Japanese diet)<br>80 75th–94th percentile<br>50 50th–74th percentile<br>25 25th–49th percentile<br>0 1st–24th percentile (Least adherence to the Japanese diet)<br><br>**Comments:** Data for the 8-item Japanese Diet Index was collected using a food frequency questionnaire based on 8 food items: rice, miso soup, seaweeds, pickles, green and yellow vegetables, fish, green tea, and red meat. For the first seven items, one point was awarded if the intake was above the median of the study population, while for red meat, one point was awarded if the intake was below the median of the study population. The total score ranged from zero to eight.
PA	**Metrics:** Total exercise time per week<br><br>**Scoring:**<br><u>Points</u> <u>Minutes</u><br>100 ≥150<br>90 120–149<br>80 90–119<br>60 60–89<br>40 30–59<br>20 1–29<br>0 0<br><br>**Comments:** During pregnancy, participants self-reported the average frequency and duration of moderate and vigorous exercise during pregnancy. One minute of moderate exercise was converted to one minute exercise and one minute of vigorous exercise was converted to two minutes of exercise. Based on this, the total exercise time per week was calculated.
Nicotine exposure	**Metrics:** Nicotine exposure status<br><br>**Scoring:**<br><u>Points</u> <u>Status</u><br>100 Never smoker<br>75 Former smoker, quit ≥5 y ago<br>50 Former smoker, quit 1–<5 y ago<br>25 Former smoker, quit <1 y ago<br>0 Current smoker<br>Subtract 20 points for non-current smokers who report secondhand smoke exposure during pregnancy.

© 2026 Ohseto H et al. *JAMA Network Open.*

	**Comments:** During pregnancy, participants self-reported current tobacco use, age of cessation, and secondhand smoke exposure during pregnancy. Inhaled nicotine-delivery system was not specifically mentioned.
Sleep health	**Metrics:** Sleep duration without naps<br><br>**Scoring:**<br>Points Hours<br>100 7–<9<br>90 9–<10<br>70 6–<7<br>40 5–<6<br>20 4–<5<br>0 <4<br>Subtract 20 points from participants with sleep apnea.<br><br>**Comments:** During pregnancy, participants self-reported total daily sleep duration including naps as categorical values (<5 h, 5-<6 h, 6-<7 h, 7-<8 h, 8-<9 h, and ≥9 h) and nap duration as continuous values. The "<5 h" category was interpreted as having a continuous value of 4.5 h, and the other categories were interpreted as having values of 5.5 h, 6.5 h, 7.5 h, 8.5 h, and 9.5 h, respectively. Subsequently, sleep duration without naps was calculated by subtracting the reported nap duration from the total sleep duration including naps. History of sleep apnea was confirmed from medical record.
BMI	**Metrics:** Pre-pregnancy BMI<br><br>**Scoring:**<br>Points kg/m^2<br>100 <23.0<br>75 23.0–24.9<br>50 25.0–29.9<br>25 30.0–34.9<br>0 ≥35.0<br><br>**Comments:** Pre-pregnancy BMI was calculated from medical record at antenatal care. The scoring system was modified from the original definition for East Asian ancestry according to the recommendations of the American Heart Association.
Blood lipids	**Metrics:** Non–HDL cholesterol<br><br>**Scoring:**<br>Points mg/dL<br>100 <130<br>60 130–159<br>40 160–189<br>20 190–219<br>0 ≥220<br>Subtract 20 points, if drug-treated level.

© 2026 Ohseto H et al. *JAMA Network Open.*

	**Comments:** Participants had blood samples taken during pregnancy. In the study population, all samples were taken before 32 weeks of gestation. Use of medications was self-reported during pregnancy.
Blood glucose	**Metrics:** Diabetes history and HbA1c<br><br>**Scoring:**<br><u>Points</u> <u>Status</u><br>100 No history of diabetes and HbA1c <5.7<br>60 No diabetes and HbA1c 5.7–6.4<br>40 Diabetes with HbA1c <7.0<br>30 Diabetes with HbA1c 7.0–7.9<br>20 Diabetes with HbA1c 8.0–8.9<br>10 Diabetes with HbA1c 9.0–9.9<br>0 Diabetes with HbA1c ≥10.0<br><br>**Comments:** Participants had blood samples taken during pregnancy. In the study population, all samples were taken before 32 weeks of gestation. History of diabetes was confirmed from self-reported use of medication and medical record.
BP	**Metrics:** Systolic and diastolic BP<br><br>**Scoring:**<br><u>Points</u> <u>mm Hg</u><br>100 <120/<80<br>75 120–129/<80<br>50 130–139 or 80–89<br>25 140–159 or 90–99<br>0 ≥160 or ≥100<br>Subtract 20 points, if drug-treated level.<br><br>**Comments:** Participants had their blood pressure measured during antenatal care. The first blood pressure measurement before 20 weeks of gestation was used to calculate the score. Use of medications was self-reported during pregnancy.

CVH: cardiovascular health: PA: physical activity; BMI: body mass index; BP: blood pressure.

© 2026 Ohseto H et al. *JAMA Network Open.*

eTable 2. Characteristics of Included and Excluded Participants in the Eligible Population

	Included<br>n = 8,238	Excluded<br>n = 10,922	P-value	Missing rate in the included population	Missing rate in the excluded population
Advanced maternal age at conception, %	2307 (28.0)	2530 (23.2)	<0.001	0.0	0.0
Educational attainment, %			<0.001	11.3	55.5
High school or lower	2223 (30.4)	1790 (36.8)			
Junior or vocational college	2905 (39.7)	1826 (37.6)			
University or higher	2181 (29.8)	1243 (25.6)			
Household income ≤ 4 million yen, %	2710 (34.2)	3645 (38.6)	<0.001	3.9	13.5
Alcohol consumption during pregnancy, %	1661 (20.2)	1895 (18.3)	0.002	0.1	5.4
Psychological distress, %	2196 (26.8)	3067 (30.8)	<0.001	0.6	8.9
Social isolation, %	1601 (19.6)	2006 (20.2)	0.290	0.7	9.1
Primipara, %	3168 (38.5)	4200 (38.5)	>0.999	0.0	0.0
Conception via IVF/ICSI, %	451 (5.5)	424 (4.1)	<0.001	0.0	5.0
Offspring's maternal and paternal family history of developmental disorders, %	13 (0.2)	15 (0.3)	0.204	10.6	55.0
Offspring sex, %			0.476	0.0	0.0
Female	4299 (52.2)	5631 (51.6)			
Male	3939 (47.8)	5290 (48.4)			
Undetermined	0 (0.0)	1 (0.0)			

© 2026 Ohseto H et al. *JAMA Network Open.*

Birth weight, g	3031.7 (394.5)	3047.6 (403.8)	0.007	0.0	0.1
Low birth weight, %	631 (7.7)	872 (8.0)	0.417	0.0	0.1
Gestational age, weeks	39.2 (1.4)	39.2 (1.4)	0.781	0.0	0.0
Preterm birth, %	387 (4.7)	514 (4.7)	>0.999	0.0	0.0
CVH score during pregnancy, point	71.4 (10.5)	69.3 (11.0)	<0.001	0.0	24.4
Postpartum CVH score, point	67.8 (10.3)	65.9 (10.9)	<0.001	37.0	68.6
Developmental delay at four years old, %					
Total	950 (11.5)	101 (13.9)	0.064	0.0	93.4
Communication	320 (3.9)	38 (4.9)	0.183	0.0	93.0
Gross motor	328 (4.0)	36 (4.7)	0.418	0.0	92.9
Fine motor	401 (4.9)	41 (5.3)	0.630	0.0	93.0
Problem solving	306 (3.7)	34 (4.5)	0.338	0.0	93.1
Personal-social	360 (4.4)	43 (5.5)	0.176	0.0	92.8

CVH, cardiovascular health; IVF/ICSI, *in vitro* fertilization/intracytoplasmic sperm injection; ASQ-3, Ages and Stages Questionnaire, Third Edition.

Data are presented as mean (standard deviation) for continuous variables and number (percentage) for categorical variables. P-values were calculated using the χ^2 test for categorical variables and the t test for continuous variables, as appropriate. The *eligible population* was defined after applying prespecified inclusion and exclusion criteria. *Included participants* represent those with complete data for both exposure and outcome and were analyzed in the main analysis, whereas *excluded participants* were omitted primarily due to missing exposure or outcome data. Missing rates are shown separately for the included and excluded populations to illustrate differences in data completeness across participant groups.

© 2026 Ohseto H et al. *JAMA Network Open.*

eTable 3. Characteristics of Participants With and Without Missing Exposure Data

	Not missing<br>n = 16,495	Missing<br>n = 2,665	P-value
Advanced maternal age at conception, %	4292 (26.0)	545 (20.5)	<0.001
Educational attainment, %			<0.001
High school or lower	3563 (32.3)	450 (39.6)	
Junior or vocational college	4285 (38.8)	446 (39.3)	
University or higher	3184 (28.9)	240 (21.1)	
Household income ≤ 4 million yen, %	5696 (36.2)	659 (40.9)	<0.001
Alcohol consumption during pregnancy, %	3193 (19.4)	363 (17.3)	0.023
Psychological distress, %	4695 (28.6)	568 (32.4)	0.001
Social isolation, %	3205 (19.6)	402 (23.3)	<0.001
Primipara, %	6313 (38.3)	1055 (39.6)	0.203
Conception via IVF/ICSI, %	812 (4.9)	63 (3.0)	<0.001
Offspring's maternal and paternal family history of developmental disorders, %	24 (0.2)	4 (0.3)	0.574
Offspring sex, %			0.027
Female	8574 (52.0)	1356 (50.9)	
Male	7921 (48.0)	1308 (49.1)	
Undetermined	0 (0.0)	1 (0.0)	
Birth weight, g	3044.5 (397.3)	3017.8 (415.3)	0.001
Low birth weight, %	1265 (7.7)	238 (8.9)	0.027
Gestational age, weeks	39.2 (1.4)	39.1 (1.5)	<0.001

© 2026 Ohseto H et al. *JAMA Network Open.*

Preterm birth, %	742 (4.5)	159 (6.0)	0.001
CVH score during pregnancy, point	70.3 (10.8)	NA	NA
Postpartum CVH score, point	67.1 (10.5)	66.4 (11.4)	0.089
Developmental delay at four years old, %			
Total	966 (11.7)	85 (12.0)	0.879
Communication	326 (3.9)	32 (4.5)	0.522
Gross motor	333 (4.0)	31 (4.4)	0.717
Fine motor	408 (4.9)	34 (4.8)	0.951
Problem solving	312 (3.8)	28 (3.9)	0.897
Personal-social	367 (4.4)	36 (5.1)	0.479

CVH, cardiovascular health; IVF/ICSI, *in vitro* fertilization/intracytoplasmic sperm injection; ASQ-3, Ages and Stages Questionnaire, Third Edition; NA, not applicable.

Data are presented as mean (standard deviation) for continuous variables and number (percentage) for categorical variables. P-values were calculated using the χ^2 test for categorical variables and the t test for continuous variables, as appropriate. Participants were classified according to the presence or absence of missing data on the exposure variable, defined as the maternal CVH score during pregnancy. *Not missing* indicates participants with complete exposure data, whereas *missing* indicates participants with missing exposure information.

© 2026 Ohseto H et al. *JAMA Network Open.*

eTable 4. Characteristics of Participants With and Without Missing Outcome Data

	Not missing<br>n = 8,948	Missing<br>n = 10,212	P-value
Advanced maternal age at conception, %	2469 (27.6)	2368 (23.2)	<0.001
Educational attainment, %			<0.001
High school or lower	2415 (30.6)	1598 (37.3)	
Junior or vocational college	3138 (39.8)	1593 (37.2)	
University or higher	2331 (29.6)	1093 (25.5)	
Household income ≤ 4 million yen, %	2907 (34.5)	3448 (38.6)	<0.001
Alcohol consumption during pregnancy, %	1775 (20.1)	1781 (18.3)	0.002
Psychological distress, %	2363 (27.1)	2900 (30.8)	<0.001
Social isolation, %	1727 (19.8)	1880 (20.0)	0.769
Primipara, %	3474 (38.8)	3894 (38.1)	0.333
Conception via IVF/ICSI, %	473 (5.3)	402 (4.1)	<0.001
Offspring's maternal and paternal family history of developmental disorders, %	14 (0.2)	14 (0.3)	0.151
Offspring sex, %			0.617
Female	4648 (51.9)	5282 (51.7)	
Male	4300 (48.1)	4929 (48.3)	
Undetermined	0 (0.0)	1 (0.0)	
Birth weight, g	3031.1 (395.8)	3049.3 (403.4)	0.002
Low birth weight, %	694 (7.8)	809 (7.9)	0.679
Gestational age, weeks	39.2 (1.4)	39.2 (1.4)	0.721

© 2026 Ohseto H et al. *JAMA Network Open.*

Preterm birth, %	432 (4.8)	469 (4.6)	0.463
CVH score during pregnancy, point	71.4 (10.5)	69.3 (11.0)	<0.001
Postpartum CVH score, point	67.7 (10.4)	65.8 (10.9)	<0.001
Developmental delay at four years old, %			
Total	1035 (11.6)	NA	NA
Communication	352 (3.9)	NA	NA
Gross motor	359 (4.0)	NA	NA
Fine motor	435 (4.9)	NA	NA
Problem solving	334 (3.7)	NA	NA
Personal-social	396 (4.4)	NA	NA

CVH, cardiovascular health; IVF/ICSI, *in vitro* fertilization/intracytoplasmic sperm injection; ASQ-3, Ages and Stages Questionnaire, Third Edition; NA, not applicable.

Data are presented as mean (standard deviation) for continuous variables and number (percentage) for categorical variables. P-values were calculated using the χ^2 test for categorical variables and the t test for continuous variables, as appropriate. Participants were classified according to the presence or absence of missing data on the exposure variable, defined as the maternal CVH score during pregnancy. *Not missing* indicates participants with complete outcome data, whereas *missing* indicates participants with missing outcome information.

© 2026 Ohseto H et al. *JAMA Network Open.*

eTable 5. Conditional associations Between Maternal Cardiovascular Health During Pregnancy and Offspring Developmental Delay at 4 Years

ASQ-3 domain	CVH levels during pregnancy	Adjusted RR (95% CI)	P-value	P for trend
Total	High	Reference		
	Moderate	1.29 (1.09–1.51)	0.003	
	Low	1.52 (1.08–2.13)	0.015	< 0.001
Communication	High	Reference		
	Moderate	1.39 (1.02–1.88)	0.035	
	Low	1.39 (0.73–2.67)	0.317	0.037
Gross motor	High	Reference		
	Moderate	1.46 (1.07–1.99)	0.017	
	Low	1.74 (0.93–3.26)	0.083	0.010
Fine motor	High	Reference		
	Moderate	1.43 (1.09–1.88)	0.010	
	Low	1.75 (1.02–3.01)	0.043	0.004
Problem solving	High	Reference		
	Moderate	1.51 (1.09–2.08)	0.013	
	Low	2.19 (1.24–3.88)	0.007	0.002
Personal-social	High	Reference		
	Moderate	1.43 (1.07–1.91)	0.016	
	Low	1.92 (1.10–3.35)	0.021	0.005

CVH, cardiovascular health; RR, risk ratio; CI, confidence interval; ASQ-3, Ages and Stages Questionnaire, Third Edition.

Covariate-adjusted RRs and 95% CIs were estimated using Poisson regression with robust variance. The models included maternal age at conception, educational attainment, household income, alcohol consumption, psychological distress, social isolation, parity, conception via in vitro fertilization/intracytoplasmic sperm injection, maternal and paternal family history of developmental disorders, and offspring sex. High CVH was the reference.

© 2026 Ohseto H et al. *JAMA Network Open.*

eTable 6. Associations Between Maternal Cardiovascular Health During Pregnancy and Offspring Developmental Delay at 4 Years: Inverse Probability of Selection Weighting Analysis

ASQ-3 domain	CVH levels during pregnancy	Crude			Adjusted		
		RR (95% CI)	P-value	P for trend	RR (95% CI)	P-value	P for trend
Total	High	Reference			Reference		
	Moderate	1.37 (1.17–1.62)	< 0.001		1.28 (1.08–1.51)	0.003	
	Low	1.90 (1.34–2.69)	< 0.001	< 0.001	1.50 (1.07–2.11)	0.019	0.001
Communication	High	Reference			Reference		
	Moderate	1.52 (1.12–2.05)	0.007		1.38 (1.02–1.87)	0.039	
	Low	1.89 (0.97–3.67)	0.062	0.003	1.40 (0.73–2.68)	0.310	0.040
Gross motor	High	Reference			Reference		
	Moderate	1.56 (1.15–2.11)	0.005		1.42 (1.04–1.94)	0.026	
	Low	2.23 (1.20–4.13)	0.011	0.001	1.70 (0.91–3.18)	0.097	0.016
Fine motor	High	Reference			Reference		
	Moderate	1.57 (1.19–2.06)	0.001		1.42 (1.08–1.87)	0.012	
	Low	2.28 (1.32–3.95)	0.003	< 0.001	1.72 (1.00–2.95)	0.050	0.005
Problem solving	High	Reference			Reference		
	Moderate	1.67 (1.21–2.30)	0.002		1.51 (1.09–2.09)	0.013	
	Low	3.01 (1.67–5.41)	< 0.001	< 0.001	2.20 (1.24–3.89)	0.007	0.002
Personal-social	High	Reference			Reference		
	Moderate	1.57 (1.18–2.11)	0.002		1.43 (1.07–1.91)	0.016	
	Low	2.61 (1.50–4.56)	< 0.001	< 0.001	1.94 (1.11–3.37)	0.020	0.005

ASQ-3, Ages and Stages Questionnaire, Third Edition; CVH, cardiovascular health; RR, risk ratio; CI, confidence interval.

© 2026 Ohseto H et al. *JAMA Network Open.*

Risk ratios and 95% confidence intervals were estimated using Poisson regression with robust error variance. Adjusted estimates were obtained using inverse probability of selection weighting to account for potential selection bias due to exclusion from the analytic sample. Stabilized weights were derived from a model predicting inclusion using the same covariates as in the main adjusted analyses. Maternal CVH during pregnancy was categorized as high (reference), moderate, and low.

© 2026 Ohseto H et al. *JAMA Network Open.*

eTable 7. Associations Between Maternal Cardiovascular Health During Pregnancy and Offspring Developmental Delay at 4 Years: Alternative Gestational Age Landmark Cutoffs

GA cutoff	ASQ-3 domain	CVH levels during pregnancy	Crude			Adjusted		
			RR (95% CI)	P-value	P for trend	RR (95% CI)	P-value	P for trend
20 weeks	Total	High	Reference			Reference		
		Moderate	1.45 (1.18–1.78)	< 0.001		1.27 (1.03–1.56)	0.027	
		Low	2.04 (1.25–3.35)	0.005	< 0.001	1.30 (0.74–2.27)	0.358	0.029
	Communication	High	Reference			Reference		
		Moderate	1.53 (1.06–2.20)	0.024		1.36 (0.93–2.00)	0.115	
		Low	1.67 (0.61–4.59)	0.323	0.020	1.07 (0.34–3.36)	0.906	0.155
	Gross motor	High	Reference			Reference		
		Moderate	1.59 (1.10–2.29)	0.013		1.29 (0.88–1.89)	0.199	
		Low	2.92 (1.33–6.38)	0.007	0.003	1.81 (0.75–4.33)	0.184	0.128
	Fine motor	High	Reference			Reference		
		Moderate	1.82 (1.28–2.58)	< 0.001		1.59 (1.10–2.30)	0.013	
		Low	2.75 (1.26–6.00)	0.011	< 0.001	2.14 (0.99–4.63)	0.054	0.005
	Problem solving	High	Reference			Reference		
		Moderate	1.87 (1.24–2.81)	0.003		1.51 (1.00–2.28)	0.052	
		Low	3.15 (1.34–7.41)	0.009	< 0.001	1.41 (0.51–3.94)	0.508	0.058
	Personal-social	High	Reference			Reference		
		Moderate	2.01 (1.38–2.93)	< 0.001		1.62 (1.11–2.39)	0.014	
		Low	2.28 (0.91–5.72)	0.078	< 0.001	1.62 (0.63–4.17)	0.313	0.013
28 weeks	Total	High	Reference			Reference		

© 2026 Ohseto H et al. *JAMA Network Open.*

		Moderate	1.39 (1.17–1.64)	< 0.001		1.29 (1.09–1.52)	0.003	
		Low	2.02 (1.42–2.88)	< 0.001	< 0.001	1.55 (1.10–2.19)	0.013	< 0.001
	Communication	High	Reference			Reference		
		Moderate	1.54 (1.13–2.09)	0.006		1.40 (1.03–1.91)	0.031	
		Low	2.10 (1.08–4.08)	0.029	0.002	1.53 (0.80–2.93)	0.203	0.026
	Gross motor	High	Reference			Reference		
		Moderate	1.56 (1.15–2.12)	0.004		1.43 (1.05–1.95)	0.024	
		Low	2.31 (1.22–4.37)	0.010	0.001	1.73 (0.90–3.32)	0.098	0.015
	Fine motor	High	Reference			Reference		
		Moderate	1.59 (1.20–2.09)	0.001		1.43 (1.09–1.89)	0.011	
		Low	2.60 (1.50–4.48)	< 0.001	< 0.001	1.85 (1.08–3.19)	0.026	0.004
	Problem solving	High	Reference			Reference		
		Moderate	1.64 (1.19–2.27)	0.003		1.49 (1.07–2.06)	0.017	
		Low	3.29 (1.83–5.90)	< 0.001	< 0.001	2.34 (1.32–4.13)	0.003	0.002
	Personal-social	High	Reference			Reference		
		Moderate	1.59 (1.19–2.13)	0.002		1.44 (1.07–1.93)	0.015	
		Low	2.90 (1.67–5.05)	< 0.001	< 0.001	2.08 (1.20–3.61)	0.009	0.003
34 weeks	Total	High	Reference			Reference		
		Moderate	1.40 (1.19–1.65)	< 0.001		1.31 (1.11–1.54)	0.001	
		Low	1.97 (1.40–2.77)	< 0.001	< 0.001	1.55 (1.11–2.16)	0.010	< 0.001
	Communication	High	Reference			Reference		
		Moderate	1.54 (1.14–2.09)	0.005		1.41 (1.04–1.91)	0.026	
		Low	1.83 (0.94–3.56)	0.075	0.003	1.36 (0.71–2.61)	0.350	0.032

© 2026 Ohseto H et al. *JAMA Network Open.*

Gross motor	High	Reference			Reference		
	Moderate	1.58 (1.16–2.13)	0.003		1.45 (1.07–1.97)	0.018	
	Low	2.38 (1.31–4.31)	0.004	< 0.001	1.82 (1.00–3.33)	0.051	0.009
Fine motor	High	Reference			Reference		
	Moderate	1.60 (1.22–2.11)	< 0.001		1.46 (1.11–1.92)	0.007	
	Low	2.31 (1.33–4.01)	0.003	< 0.001	1.73 (1.00–2.97)	0.048	0.003
Problem solving	High	Reference			Reference		
	Moderate	1.68 (1.21–2.31)	0.002		1.52 (1.10–2.11)	0.011	
	Low	2.93 (1.63–5.27)	< 0.001	< 0.001	2.14 (1.21–3.79)	0.009	0.002
Personal-social	High	Reference			Reference		
	Moderate	1.65 (1.23–2.21)	< 0.001		1.50 (1.12–2.01)	0.007	
	Low	2.64 (1.51–4.61)	< 0.001	< 0.001	1.95 (1.12–3.41)	0.019	0.002

ASQ-3, Ages and Stages Questionnaire, Third Edition; CVH, cardiovascular health; GA, gestational age; RR, risk ratio; CI, confidence interval.

Risk ratios and 95% confidence intervals were estimated using Poisson regression models with robust error variance. Landmark analyses were conducted by redefining the gestational age cutoff for CVH assessment at 20, 28, and 34 weeks of gestation. Maternal CVH during pregnancy was categorized as high (reference), moderate, and low.

© 2026 Ohseto H et al. *JAMA Network Open.*

eTable 8. Associations Between Maternal Cardiovascular Health During Pregnancy and Offspring Developmental Delay at 4 Years Among Term Births

ASQ-3 domain	CVH levels during pregnancy	Crude RR (95% CI)	Crude P-value	Crude P for trend	Adjusted RR (95% CI)	Adjusted P-value	Adjusted P for trend
Total	High	Reference			Reference		
	Moderate	1.38 (1.18–1.61)	< 0.001		1.29 (1.10–1.50)	0.001	
	Low	1.71 (1.23–2.38)	0.001	< 0.001	1.35 (0.98–1.87)	0.066	0.001
Communication	High	Reference			Reference		
	Moderate	1.54 (1.13–2.09)	0.006		1.39 (1.03–1.89)	0.034	
	Low	1.41 (0.67–2.94)	0.362	0.007	0.99 (0.48–2.02)	0.971	0.099
Gross motor	High	Reference			Reference		
	Moderate	1.46 (1.16–1.82)	0.001		1.36 (1.08–1.70)	0.009	
	Low	1.65 (1.00–2.73)	0.049	< 0.001	1.34 (0.81–2.21)	0.262	0.012
Fine motor	High	Reference			Reference		
	Moderate	1.61 (1.22–2.13)	< 0.001		1.46 (1.10–1.93)	0.008	
	Low	2.26 (1.30–3.92)	0.004	< 0.001	1.62 (0.94–2.81)	0.083	0.005
Problem solving	High	Reference			Reference		
	Moderate	1.75 (1.25–2.44)	< 0.001		1.59 (1.14–2.22)	0.006	
	Low	2.76 (1.50–5.08)	0.001	< 0.001	1.95 (1.08–3.54)	0.028	0.002
Personal-social	High	Reference			Reference		
	Moderate	1.72 (1.27–2.32)	< 0.001		1.57 (1.16–2.11)	0.003	
	Low	2.58 (1.47–4.53)	< 0.001	< 0.001	1.86 (1.05–3.28)	0.033	0.001

ASQ-3, Ages and Stages Questionnaire, Third Edition; CVH, cardiovascular health; RR, risk ratio; CI, confidence interval.

© 2026 Ohseto H et al. *JAMA Network Open.*

Risk ratios and 95% confidence intervals were estimated using Poisson regression models with robust error variance. Analyses were restricted to term births (gestational age ≥37 weeks). Maternal CVH during pregnancy was categorized as high (reference), moderate, and low.

© 2026 Ohseto H et al. *JAMA Network Open.*

eTable 9. Associations Between Maternal Cardiovascular Health During Pregnancy and Offspring Developmental Delay at 4 Years After Additional Adjustment for Gestational Age at Delivery

ASQ-3 domain	CVH levels during pregnancy	GA-adjusted RR (95% CI)	GA-adjusted P-value	GA-adjusted P for trend
Total	High	Reference		
	Moderate	1.28 (1.09–1.51)	0.003	
	Low	1.51 (1.08–2.11)	0.017	0.001
Communication	High	Reference		
	Moderate	1.38 (1.02–1.88)	0.036	
	Low	1.38 (0.73–2.64)	0.325	0.039
Gross motor	High	Reference		
	Moderate	1.45 (1.07–1.98)	0.018	
	Low	1.72 (0.92–3.22)	0.091	0.011
Fine motor	High	Reference		
	Moderate	1.43 (1.09–1.88)	0.010	
	Low	1.74 (1.02–3.00)	0.044	0.004
Problem solving	High	Reference		
	Moderate	1.51 (1.09–2.08)	0.013	
	Low	2.19 (1.24–3.88)	0.007	0.002
Personal-social	High	Reference		
	Moderate	1.43 (1.07–1.91)	0.017	
	Low	1.92 (1.10–3.35)	0.022	0.005

ASQ-3, Ages and Stages Questionnaire, Third Edition; CVH, cardiovascular health; GA, gestational age; RR, risk ratio; CI, confidence interval.

Risk ratios and 95% confidence intervals were estimated using Poisson regression models with robust error variance. Models were additionally adjusted for gestational age at delivery.

© 2026 Ohseto H et al. *JAMA Network Open.*

eTable 10. Associations Between Maternal Cardiovascular Health During Pregnancy and Offspring Developmental Delay at 4 Years: Leave-One-Component-Out Analysis

ASQ-3 domain	Excluded CVH component	CVH levels during pregnancy	Crude			Adjusted		
			RR (95% CI)	P-value	P for trend	RR (95% CI)	P-value	P for trend
Total	Diet	High	Reference			Reference		
		Moderate	1.20 (1.05–1.38)	0.006		1.12 (0.98–1.28)	0.087	
		Low	1.58 (1.06–2.36)	0.024	0.002	1.27 (0.86–1.87)	0.231	0.061
	PA	High	Reference			Reference		
		Moderate	1.32 (1.17–1.49)	< 0.001		1.21 (1.07–1.37)	0.002	
		Low	1.71 (1.08–2.69)	0.022	< 0.001	1.35 (0.87–2.09)	0.176	0.001
	Nicotine exposure	High	Reference			Reference		
		Moderate	1.54 (1.29–1.84)	< 0.001		1.47 (1.23–1.76)	< 0.001	
		Low	2.14 (1.53–2.99)	< 0.001	< 0.001	1.79 (1.29–2.49)	< 0.001	< 0.001
	Sleep health	High	Reference			Reference		
		Moderate	1.29 (1.10–1.51)	0.002		1.20 (1.03–1.41)	0.022	
		Low	1.81 (1.35–2.43)	< 0.001	< 0.001	1.49 (1.12–2.00)	0.007	0.004
	BMI	High	Reference			Reference		
		Moderate	1.49 (1.23–1.81)	< 0.001		1.40 (1.16–1.70)	< 0.001	
		Low	1.96 (1.44–2.67)	< 0.001	< 0.001	1.52 (1.12–2.07)	0.008	< 0.001
	Blood lipids	High	Reference			Reference		
		Moderate	1.35 (1.15–1.59)	< 0.001		1.27 (1.07–1.49)	0.005	
		Low	1.91 (1.39–2.63)	< 0.001	< 0.001	1.50 (1.09–2.06)	0.012	0.001
	Blood glucose	High	Reference			Reference		

© 2026 Ohseto H et al. *JAMA Network Open.*

		Moderate	1.55 (1.26–1.91)	< 0.001		1.46 (1.19–1.79)	< 0.001	
		Low	1.93 (1.47–2.54)	< 0.001	< 0.001	1.55 (1.18–2.04)	0.002	< 0.001
	BP	High	Reference			Reference		
		Moderate	1.52 (1.25–1.84)	< 0.001		1.42 (1.17–1.72)	< 0.001	
		Low	1.77 (1.32–2.39)	< 0.001	< 0.001	1.41 (1.04–1.89)	0.025	0.001
Communication	Diet	High	Reference			Reference		
		Moderate	1.39 (1.09–1.79)	0.009		1.28 (1.00–1.64)	0.048	
		Low	1.90 (0.94–3.85)	0.074	0.004	1.46 (0.73–2.92)	0.290	0.039
	PA	High	Reference			Reference		
		Moderate	1.28 (1.03–1.59)	0.027		1.16 (0.93–1.45)	0.183	
		Low	1.87 (0.85–4.13)	0.121	0.014	1.44 (0.67–3.09)	0.351	0.139
	Nicotine exposure	High	Reference			Reference		
		Moderate	1.67 (1.20–2.31)	0.002		1.56 (1.13–2.16)	0.008	
		Low	2.15 (1.14–4.03)	0.017	< 0.001	1.66 (0.90–3.08)	0.105	0.005
	Sleep health	High	Reference			Reference		
		Moderate	1.16 (0.88–1.52)	0.305		1.06 (0.81–1.40)	0.659	
		Low	2.08 (1.27–3.39)	0.003	0.038	1.66 (1.02–2.70)	0.043	0.184
	BMI	High	Reference			Reference		
		Moderate	1.71 (1.19–2.45)	0.004		1.56 (1.09–2.25)	0.015	
		Low	2.27 (1.29–3.99)	0.005	< 0.001	1.69 (0.96–2.98)	0.069	0.013
	Blood lipids	High	Reference			Reference		
		Moderate	1.69 (1.24–2.32)	0.001		1.55 (1.13–2.13)	0.006	
		Low	2.04 (1.10–3.82)	0.025	< 0.001	1.54 (0.83–2.87)	0.170	0.007

© 2026 Ohseto H et al. *JAMA Network Open.*

	Blood glucose	High	Reference			Reference		
		Moderate	1.94 (1.29–2.91)	0.001		1.79 (1.20–2.69)	0.005	
		Low	2.42 (1.44–4.07)	< 0.001	< 0.001	1.88 (1.12–3.16)	0.016	0.005
	BP	High	Reference			Reference		
		Moderate	1.95 (1.33–2.87)	< 0.001		1.80 (1.23–2.64)	0.003	
		Low	2.44 (1.41–4.22)	0.001	< 0.001	1.87 (1.08–3.22)	0.024	0.002
Gross motor	Diet	High	Reference			Reference		
		Moderate	1.41 (1.11–1.81)	0.006		1.31 (1.02–1.68)	0.032	
		Low	2.11 (1.09–4.11)	0.028	0.002	1.65 (0.84–3.26)	0.146	0.020
	PA	High	Reference			Reference		
		Moderate	1.37 (1.10–1.71)	0.004		1.27 (1.02–1.59)	0.031	
		Low	1.90 (0.86–4.20)	0.112	0.002	1.49 (0.67–3.32)	0.329	0.025
	Nicotine exposure	High	Reference			Reference		
		Moderate	1.81 (1.30–2.53)	< 0.001		1.68 (1.20–2.36)	0.002	
		Low	2.45 (1.33–4.53)	0.004	< 0.001	1.97 (1.06–3.67)	0.032	0.001
	Sleep health	High	Reference			Reference		
		Moderate	1.33 (1.00–1.77)	0.048		1.23 (0.92–1.63)	0.157	
		Low	2.15 (1.30–3.56)	0.003	0.006	1.75 (1.05–2.94)	0.033	0.044
	BMI	High	Reference			Reference		
		Moderate	1.49 (1.06–2.08)	0.022		1.36 (0.96–1.91)	0.079	
		Low	2.07 (1.21–3.55)	0.008	0.004	1.60 (0.92–2.77)	0.096	0.048
	Blood lipids	High	Reference			Reference		
		Moderate	1.45 (1.08–1.95)	0.014		1.33 (0.98–1.79)	0.066	

© 2026 Ohseto H et al. *JAMA Network Open.*

		Low	2.21 (1.26–3.86)	0.006	0.002	1.69 (0.95–3.01)	0.072	0.032
	Blood glucose	High	Reference			Reference		
		Moderate	1.68 (1.15–2.46)	0.007		1.54 (1.06–2.26)	0.025	
		Low	2.22 (1.36–3.63)	0.001	< 0.001	1.77 (1.08–2.92)	0.025	0.014
	BP	High	Reference			Reference		
		Moderate	1.80 (1.25–2.60)	0.002		1.65 (1.14–2.38)	0.008	
		Low	2.10 (1.22–3.62)	0.007	< 0.001	1.63 (0.94–2.84)	0.084	0.013
Fine motor	Diet	High	Reference			Reference		
		Moderate	1.50 (1.19–1.87)	< 0.001		1.38 (1.10–1.72)	0.005	
		Low	2.41 (1.36–4.28)	0.003	< 0.001	1.90 (1.07–3.37)	0.028	0.002
	PA	High	Reference			Reference		
		Moderate	1.50 (1.23–1.83)	< 0.001		1.35 (1.11–1.65)	0.003	
		Low	1.91 (0.92–3.97)	0.081	< 0.001	1.46 (0.72–2.96)	0.295	0.002
	Nicotine exposure	High	Reference			Reference		
		Moderate	1.82 (1.34–2.46)	< 0.001		1.71 (1.26–2.31)	< 0.001	
		Low	2.96 (1.77–4.95)	< 0.001	< 0.001	2.31 (1.38–3.87)	0.001	< 0.001
	Sleep health	High	Reference			Reference		
		Moderate	1.36 (1.05–1.76)	0.020		1.24 (0.96–1.61)	0.098	
		Low	2.36 (1.52–3.68)	< 0.001	< 0.001	1.85 (1.19–2.87)	0.006	0.013
	BMI	High	Reference			Reference		
		Moderate	1.85 (1.32–2.58)	< 0.001		1.70 (1.22–2.37)	0.002	
		Low	2.83 (1.74–4.61)	< 0.001	< 0.001	2.03 (1.24–3.31)	0.005	< 0.001
	Blood lipids	High	Reference			Reference		

© 2026 Ohseto H et al. *JAMA Network Open.*

		Moderate	1.54 (1.17–2.02)	0.002		1.41 (1.07–1.86)	0.014	
		Low	2.54 (1.56–4.14)	< 0.001	< 0.001	1.87 (1.15–3.04)	0.011	0.003
	Blood glucose	High	Reference			Reference		
		Moderate	1.92 (1.33–2.76)	< 0.001		1.78 (1.24–2.55)	0.002	
		Low	2.90 (1.86–4.52)	< 0.001	< 0.001	2.19 (1.40–3.42)	< 0.001	< 0.001
	BP	High	Reference			Reference		
		Moderate	2.01 (1.42–2.84)	< 0.001		1.85 (1.31–2.61)	< 0.001	
		Low	2.74 (1.70–4.42)	< 0.001	< 0.001	2.06 (1.27–3.32)	0.003	< 0.001
Problem solving	Diet	High	Reference			Reference		
		Moderate	1.48 (1.15–1.92)	0.003		1.35 (1.04–1.75)	0.023	
		Low	2.90 (1.57–5.33)	< 0.001	< 0.001	2.13 (1.16–3.91)	0.014	0.007
	PA	High	Reference			Reference		
		Moderate	1.55 (1.23–1.95)	< 0.001		1.38 (1.09–1.74)	0.007	
		Low	3.33 (1.74–6.37)	< 0.001	< 0.001	2.41 (1.30–4.47)	0.005	0.001
	Nicotine exposure	High	Reference			Reference		
		Moderate	1.87 (1.32–2.67)	< 0.001		1.79 (1.26–2.54)	0.001	
		Low	3.37 (1.89–6.00)	< 0.001	< 0.001	2.70 (1.54–4.73)	< 0.001	< 0.001
	Sleep health	High	Reference			Reference		
		Moderate	1.36 (1.01–1.84)	0.041		1.25 (0.93–1.68)	0.142	
		Low	2.36 (1.41–3.94)	0.001	0.003	1.81 (1.10–3.00)	0.020	0.031
	BMI	High	Reference			Reference		
		Moderate	1.72 (1.18–2.50)	0.005		1.59 (1.09–2.31)	0.016	
		Low	2.82 (1.64–4.86)	< 0.001	< 0.001	2.03 (1.17–3.51)	0.011	0.004

© 2026 Ohseto H et al. *JAMA Network Open.*

	Blood lipids	High	Reference			Reference		
		Moderate	1.74 (1.25–2.40)	< 0.001		1.58 (1.14–2.20)	0.006	
		Low	2.38 (1.29–4.37)	0.005	< 0.001	1.72 (0.94–3.15)	0.076	0.004
	Blood glucose	High	Reference			Reference		
		Moderate	1.91 (1.26–2.89)	0.002		1.77 (1.17–2.68)	0.007	
		Low	2.61 (1.55–4.38)	< 0.001	< 0.001	1.94 (1.15–3.26)	0.013	0.005
	BP	High	Reference			Reference		
		Moderate	2.09 (1.39–3.14)	< 0.001		1.92 (1.28–2.88)	0.002	
		Low	2.86 (1.64–4.98)	< 0.001	< 0.001	2.09 (1.21–3.64)	0.009	< 0.001
Personal-social	Diet	High	Reference			Reference		
		Moderate	1.49 (1.17–1.88)	< 0.001		1.35 (1.07–1.71)	0.013	
		Low	1.09 (0.45–2.65)	0.841	0.002	0.81 (0.34–1.95)	0.643	0.039
	PA	High	Reference			Reference		
		Moderate	1.39 (1.13–1.71)	0.002		1.25 (1.02–1.54)	0.035	
		Low	0.87 (0.28–2.67)	0.801	0.003	0.64 (0.21–1.89)	0.416	0.086
	Nicotine exposure	High	Reference			Reference		
		Moderate	1.79 (1.30–2.46)	< 0.001		1.68 (1.22–2.30)	0.001	
		Low	2.73 (1.56–4.77)	< 0.001	< 0.001	2.11 (1.21–3.68)	0.009	< 0.001
	Sleep health	High	Reference			Reference		
		Moderate	1.27 (0.97–1.65)	0.081		1.15 (0.88–1.50)	0.295	
		Low	1.56 (0.92–2.65)	0.102	0.042	1.19 (0.70–2.02)	0.530	0.302
	BMI	High	Reference			Reference		
		Moderate	1.81 (1.27–2.56)	< 0.001		1.67 (1.17–2.36)	0.004	

© 2026 Ohseto H et al. *JAMA Network Open.*

	Low	2.61 (1.55–4.40)	< 0.001	< 0.001	1.89 (1.11–3.21)	0.019	0.002
Blood lipids	High	Reference			Reference		
	Moderate	1.55 (1.16–2.07)	0.003		1.41 (1.06–1.88)	0.020	
	Low	2.26 (1.32–3.89)	0.003	< 0.001	1.65 (0.95–2.86)	0.073	0.011
Blood glucose	High	Reference			Reference		
	Moderate	2.03 (1.37–2.99)	< 0.001		1.87 (1.27–2.76)	0.002	
	Low	2.53 (1.55–4.14)	< 0.001	< 0.001	1.90 (1.16–3.12)	0.011	0.003
BP	High	Reference			Reference		
	Moderate	1.80 (1.27–2.55)	< 0.001		1.65 (1.16–2.33)	0.005	
	Low	2.19 (1.32–3.65)	0.003	< 0.001	1.62 (0.97–2.72)	0.066	0.009

ASQ-3, Ages and Stages Questionnaire, Third Edition; CVH, cardiovascular health; LE8, Life's Essential 8; PA, physical activity; BMI, body mass index; BP, blood pressure; RR, risk ratio; CI, confidence interval.

Risk ratios and 95% confidence intervals were estimated using Poisson regression models with robust error variance. For each analysis, the CVH score during pregnancy was recalculated using seven LE8 components, excluding the component indicated in the table, and associations were re-estimated using these alternative composite scores. Maternal CVH during pregnancy was categorized as high (reference), moderate, and low.

© 2026 Ohseto H et al. *JAMA Network Open.*

eTable 11. Associations Between Maternal Cardiovascular Health During Pregnancy and Offspring Developmental Delay at 4 Years Using the DASH Score for the Dietary Component

ASQ-3 domain	CVH levels during pregnancy	Crude			Adjusted		
		RR (95% CI)	P-value	P for trend	RR (95% CI)	P-value	P for trend
Total	High	Reference			Reference		
	Moderate	1.43 (1.20–1.69)	< 0.001		1.35 (1.14–1.60)	< 0.001	
	Low	2.01 (1.44–2.81)	0.003	< 0.001	1.64 (1.18–2.28)	< 0.001	< 0.001
Communication	High	Reference			Reference		
	Moderate	1.70 (1.23–2.36)	0.005		1.60 (1.16–2.21)	0.001	
	Low	2.06 (1.07–3.95)	0.129	< 0.001	1.65 (0.86–3.13)	0.029	0.004
Gross motor	High	Reference			Reference		
	Moderate	1.37 (1.02–1.85)	0.098		1.29 (0.95–1.74)	0.038	
	Low	2.30 (1.32–4.03)	0.031	0.006	1.87 (1.06–3.31)	0.003	0.031
Fine motor	High	Reference			Reference		
	Moderate	1.77 (1.31–2.37)	< 0.001		1.66 (1.23–2.22)	< 0.001	
	Low	3.13 (1.90–5.17)	< 0.001	< 0.001	2.51 (1.52–4.14)	< 0.001	< 0.001
Problem solving	High	Reference			Reference		
	Moderate	1.88 (1.33–2.67)	0.002		1.73 (1.22–2.46)	< 0.001	
	Low	3.95 (2.28–6.85)	< 0.001	< 0.001	2.98 (1.73–5.16)	< 0.001	< 0.001
Personal-social	High	Reference			Reference		
	Moderate	1.54 (1.15–2.07)	0.019		1.43 (1.06–1.92)	0.004	
	Low	2.15 (1.21–3.82)	0.085	< 0.001	1.67 (0.93–2.98)	0.009	0.012

ASQ-3, Ages and Stages Questionnaire, Third Edition; CVH, cardiovascular health; DASH, Dietary Approaches to Stop Hypertension; RR, risk ratio; CI,

confidence interval.

Risk ratios and 95% confidence intervals were estimated using Poisson regression models with robust error variance. In this sensitivity analysis, the dietary component of maternal CVH during pregnancy was defined using the DASH score instead of the Japanese Diet Index, while all other CVH components and model specifications remained unchanged. Maternal CVH during pregnancy was categorized as high (reference), moderate, and low.

© 2026 Ohseto H et al. *JAMA Network Open.*

eTable 12. Associations Between Individual Maternal Cardiovascular Health Components During Pregnancy and Offspring Developmental Delay at 4 Years

ASQ-3 domain	CVH component	CVH levels during pregnancy	Crude RR (95% CI)	Crude P-value	Adjusted RR (95% CI)	Adjusted P-value
Total	Diet	High	Reference		Reference	
		Moderate/Low	1.19 (1.05–1.34)	0.007	1.13 (1.00–1.27)	0.057
	PA	High	Reference		Reference	
		Moderate/Low	1.10 (0.95–1.28)	0.204	1.10 (0.95–1.28)	0.216
	Nicotine exposure	High	Reference		Reference	
		Moderate/Low	0.93 (0.82–1.06)	0.270	0.84 (0.74–0.96)	0.010
	Sleep health	High	Reference		Reference	
		Moderate/Low	1.08 (0.95–1.22)	0.235	1.04 (0.93–1.18)	0.484
	BMI	High	Reference		Reference	
		Moderate/Low	1.22 (1.06–1.40)	0.004	1.14 (1.00–1.31)	0.047
	Blood lipids	High	Reference		Reference	
		Moderate/Low	1.03 (0.92–1.17)	0.600	1.01 (0.89–1.14)	0.891
	Blood glucose	High	Reference		Reference	
		Moderate/Low	1.25 (0.80–1.95)	0.318	1.24 (0.80–1.94)	0.338
	BP	High	Reference		Reference	
		Moderate/Low	1.20 (1.06–1.37)	0.006	1.13 (1.00–1.29)	0.059
Communication	Diet	High	Reference		Reference	
		Moderate/Low	1.18 (0.94–1.47)	0.156	1.10 (0.88–1.38)	0.401
	PA	High	Reference		Reference	

© 2026 Ohseto H et al. *JAMA Network Open.*

		Moderate/Low	1.32 (0.99–1.76)	0.057	1.31 (0.98–1.75)	0.071
	Nicotine exposure	High	Reference		Reference	
		Moderate/Low	0.99 (0.79–1.24)	0.908	0.91 (0.72–1.15)	0.429
	Sleep health	High	Reference		Reference	
		Moderate/Low	0.97 (0.78–1.20)	0.764	0.92 (0.74–1.14)	0.444
	BMI	High	Reference		Reference	
		Moderate/Low	1.36 (1.08–1.73)	0.010	1.27 (1.00–1.61)	0.046
	Blood lipids	High	Reference		Reference	
		Moderate/Low	0.98 (0.79–1.21)	0.844	0.95 (0.76–1.18)	0.615
	Blood glucose	High	Reference		Reference	
		Moderate/Low	0.65 (0.21–2.00)	0.454	0.64 (0.20–1.98)	0.434
	BP	High	Reference		Reference	
		Moderate/Low	1.01 (0.79–1.29)	0.953	0.93 (0.73–1.18)	0.555
Gross motor	Diet	High	Reference		Reference	
		Moderate/Low	1.13 (0.91–1.41)	0.276	1.06 (0.85–1.32)	0.586
	PA	High	Reference		Reference	
		Moderate/Low	1.18 (0.89–1.54)	0.246	1.15 (0.88–1.51)	0.315
	Nicotine exposure	High	Reference		Reference	
		Moderate/Low	0.82 (0.65–1.03)	0.082	0.75 (0.59–0.95)	0.019
	Sleep health	High	Reference		Reference	
		Moderate/Low	1.15 (0.92–1.43)	0.220	1.11 (0.89–1.37)	0.364
	BMI	High	Reference		Reference	
		Moderate/Low	1.28 (1.01–1.62)	0.043	1.21 (0.96–1.54)	0.109

© 2026 Ohseto H et al. *JAMA Network Open.*

	Blood lipids	High	Reference		Reference	
		Moderate/Low	1.10 (0.89–1.37)	0.379	1.07 (0.87–1.33)	0.515
	Blood glucose	High	Reference		Reference	
		Moderate/Low	1.94 (1.03–3.67)	0.041	1.85 (0.96–3.57)	0.067
	BP	High	Reference		Reference	
		Moderate/Low	1.14 (0.90–1.44)	0.279	1.07 (0.84–1.35)	0.581
Fine motor	Diet	High	Reference		Reference	
		Moderate/Low	1.24 (1.01–1.51)	0.036	1.13 (0.93–1.38)	0.232
	PA	High	Reference		Reference	
		Moderate/Low	1.25 (0.97–1.60)	0.083	1.23 (0.95–1.58)	0.115
	Nicotine exposure	High	Reference		Reference	
		Moderate/Low	1.04 (0.85–1.26)	0.734	0.94 (0.77–1.15)	0.551
	Sleep health	High	Reference		Reference	
		Moderate/Low	1.15 (0.94–1.40)	0.164	1.10 (0.91–1.34)	0.323
	BMI	High	Reference		Reference	
		Moderate/Low	1.41 (1.15–1.74)	0.001	1.31 (1.06–1.61)	0.011
	Blood lipids	High	Reference		Reference	
		Moderate/Low	0.98 (0.81–1.19)	0.860	0.96 (0.79–1.16)	0.642
	Blood glucose	High	Reference		Reference	
		Moderate/Low	1.58 (0.84–2.98)	0.158	1.76 (0.94–3.31)	0.077
	BP	High	Reference		Reference	
		Moderate/Low	1.24 (1.01–1.53)	0.040	1.15 (0.93–1.41)	0.189
Problem solving	Diet	High	Reference		Reference	

© 2026 Ohseto H et al. *JAMA Network Open.*

		Moderate/Low	1.16 (0.92–1.45)	0.212	1.07 (0.85–1.35)	0.547
	PA	High	Reference		Reference	
		Moderate/Low	1.08 (0.82–1.42)	0.590	1.09 (0.83–1.44)	0.530
	Nicotine exposure	High	Reference		Reference	
		Moderate/Low	0.97 (0.77–1.22)	0.809	0.83 (0.65–1.05)	0.123
	Sleep health	High	Reference		Reference	
		Moderate/Low	1.15 (0.92–1.45)	0.215	1.10 (0.88–1.38)	0.401
	BMI	High	Reference		Reference	
		Moderate/Low	1.54 (1.22–1.96)	< 0.001	1.41 (1.12–1.78)	0.004
	Blood lipids	High	Reference		Reference	
		Moderate/Low	1.10 (0.88–1.38)	0.395	1.07 (0.86–1.35)	0.533
	Blood glucose	High	Reference		Reference	
		Moderate/Low	2.09 (1.10–3.95)	0.024	2.10 (1.11–3.97)	0.023
	BP	High	Reference		Reference	
		Moderate/Low	1.11 (0.87–1.41)	0.419	1.02 (0.80–1.31)	0.846
Personal-social	Diet	High	Reference		Reference	
		Moderate/Low	1.14 (0.93–1.41)	0.207	1.07 (0.87–1.32)	0.515
	PA	High	Reference		Reference	
		Moderate/Low	1.07 (0.83–1.38)	0.579	1.04 (0.81–1.34)	0.756
	Nicotine exposure	High	Reference		Reference	
		Moderate/Low	0.87 (0.70–1.08)	0.208	0.76 (0.61–0.95)	0.018
	Sleep health	High	Reference		Reference	
		Moderate/Low	1.19 (0.97–1.47)	0.098	1.14 (0.93–1.41)	0.211

© 2026 Ohseto H et al. *JAMA Network Open.*

BMI	High	Reference		Reference	
	Moderate/Low	1.34 (1.07–1.67)	0.011	1.22 (0.98–1.53)	0.076
Blood lipids	High	Reference		Reference	
	Moderate/Low	0.98 (0.80–1.21)	0.877	0.95 (0.78–1.17)	0.639
Blood glucose	High	Reference		Reference	
	Moderate/Low	1.56 (0.79–3.08)	0.195	1.54 (0.77–3.07)	0.221
BP	High	Reference		Reference	
	Moderate/Low	1.23 (0.99–1.54)	0.062	1.14 (0.92–1.42)	0.235

ASQ-3, Ages and Stages Questionnaire, Third Edition; CVH, cardiovascular health; PA, physical activity; BMI, body mass index; BP, blood pressure; RR, risk ratio; CI, confidence interval.

Risk ratios and 95% confidence intervals were estimated using Poisson regression models with robust error variance. Each CVH component during pregnancy was dichotomized into high versus moderate/low categories and examined separately. High category was used as the reference. Adjusted models included the same covariates as in the main analyses.

© 2026 Ohseto H et al. *JAMA Network Open.*

eTable 13. Joint Associations of Maternal Cardiovascular Health During Pregnancy and Postpartum With Offspring Developmental Delay at 4 Years

ASQ-3 domain	CVH levels during pregnancy	Postpartum CVH levels	Crude RR (95% CI)	P-value	Adjusted RR (95% CI)	P-value
Total	High	High	Reference		Reference	
		Moderate/Low	1.20 (0.73–1.96)	0.469	1.17 (0.72–1.90)	0.513
	Moderate/Low	High	1.31 (0.76–2.26)	0.332	1.26 (0.74–2.15)	0.401
		Moderate/Low	1.63 (1.03–2.56)	0.035	1.42 (0.91–2.22)	0.127
Communication	High	High	Reference		Reference	
		Moderate/Low	1.05 (0.44–2.54)	0.910	1.01 (0.42–2.46)	0.975
	Moderate/Low	High	0.90 (0.32–2.55)	0.840	0.85 (0.30–2.42)	0.761
		Moderate/Low	1.74 (0.78–3.89)	0.177	1.47 (0.66–3.29)	0.349
Gross motor	High	High	Reference		Reference	
		Moderate/Low	0.82 (0.38–1.80)	0.624	0.79 (0.36–1.72)	0.556
	Moderate/Low	High	1.18 (0.50–2.76)	0.706	1.09 (0.47–2.54)	0.835
		Moderate/Low	1.22 (0.61–2.46)	0.570	1.03 (0.51–2.07)	0.936
Fine motor	High	High	Reference		Reference	
		Moderate/Low	2.72 (0.84–8.81)	0.096	2.56 (0.80–8.23)	0.115
	Moderate/Low	High	3.82 (1.13–12.88)	0.031	3.52 (1.05–11.81)	0.042
		Moderate/Low	4.06 (1.31–12.59)	0.015	3.38 (1.10–10.45)	0.034
Problem solving	High	High	Reference		Reference	
		Moderate/Low	0.89 (0.33–2.40)	0.824	0.86 (0.33–2.29)	0.767
	Moderate/Low	High	1.21 (0.41–3.57)	0.727	1.14 (0.39–3.32)	0.812

© 2026 Ohseto H et al. *JAMA Network Open.*

		Moderate/Low	2.02 (0.84–4.88)	0.117	1.71 (0.71–4.11)	0.231
Personal-social	High	High	Reference		Reference	
		Moderate/Low	0.79 (0.36–1.73)	0.555	0.76 (0.35–1.65)	0.482
	Moderate/Low	High	1.09 (0.46–2.60)	0.839	1.01 (0.42–2.40)	0.986
		Moderate/Low	1.51 (0.75–3.03)	0.243	1.25 (0.62–2.49)	0.533

ASQ-3, Ages and Stages Questionnaire, Third Edition; CVH, cardiovascular health; RR, risk ratio; CI, confidence interval.

Risk ratios and 95% confidence intervals were estimated using Poisson regression models with robust error variance. Maternal CVH during pregnancy and postpartum was dichotomized as high versus moderate/low and jointly classified into four exposure groups. High CVH during both pregnancy and postpartum was used as the reference category. Adjusted models included the same covariates as in the main analyses.

© 2026 Ohseto H et al. *JAMA Network Open.*

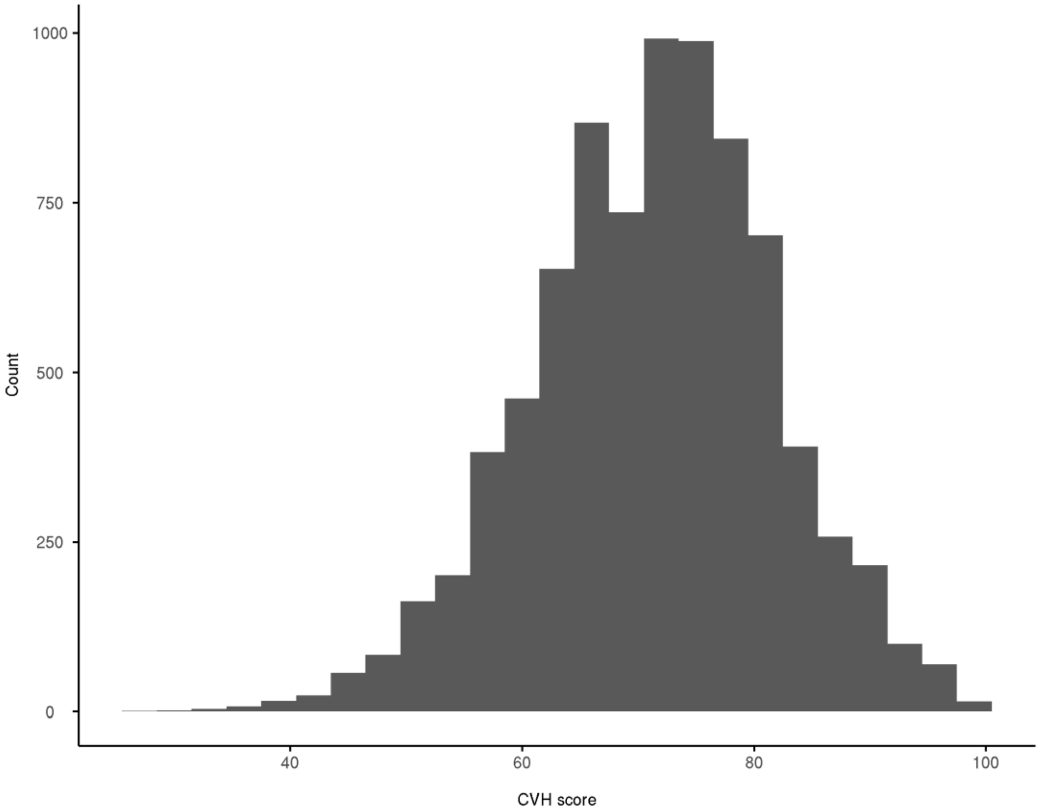

eFigure 1. Distribution of Maternal Cardiovascular Health Scores During Pregnancy

CVH scores during pregnancy were calculated based on Life's Essential 8 metrics and ranged from 0 to 100, with higher scores indicating more favorable cardiovascular health. The histogram displays the distribution of CVH scores in the study population.

© 2026 Ohseto H et al. *JAMA Network Open.*

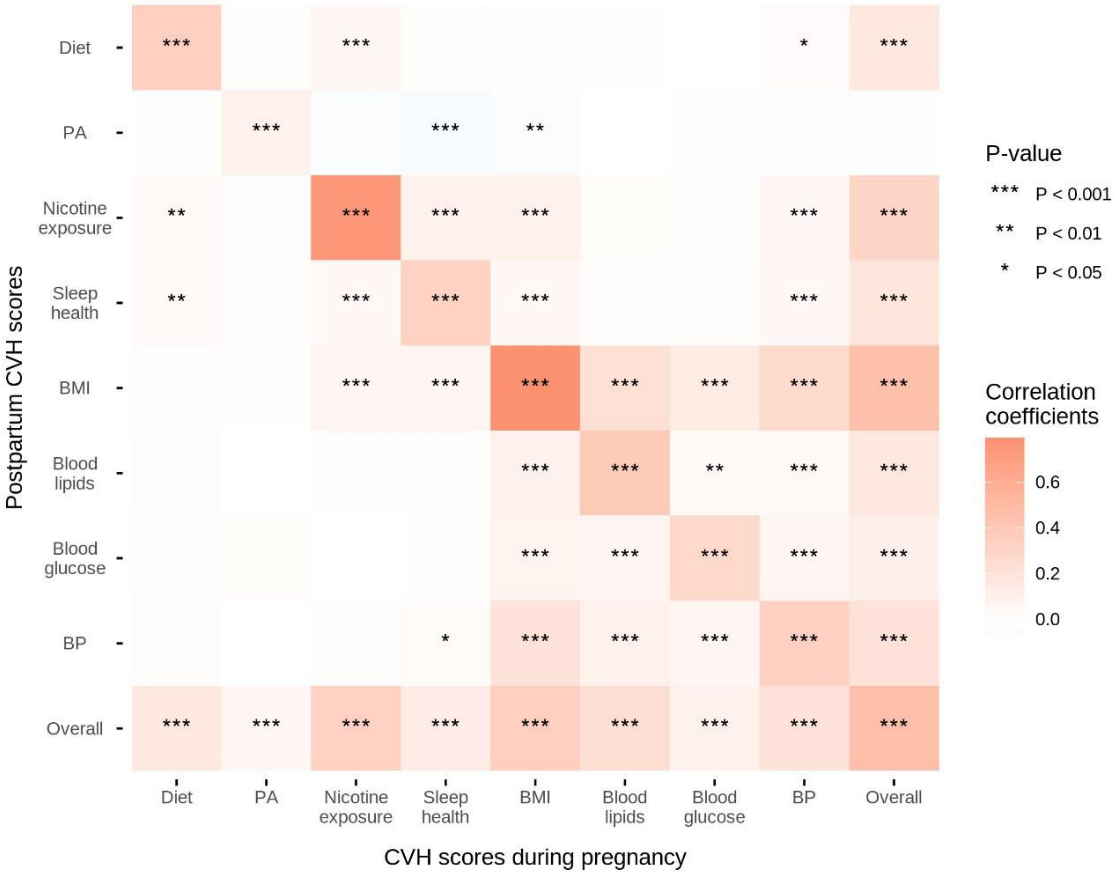

eFigure 2. Heatmap of Correlations Between Cardiovascular Health Components During Pregnancy and Postpartum

The heatmap represents Pearson's correlation coefficients between the component CVH scores during pregnancy and postpartum. Red, blue, and white indicate positive, negative, and no association, respectively. Darker colors indicate stronger associations. The asterisk indicates the P-value.

CVH, cardiovascular health; PA, physical activity; BMI, body mass index; BP, blood pressure.

© 2026 Ohseto H et al. *JAMA Network Open.*
