# Supplementary material for: Maternal Cardiovascular Health During Pregnancy and Offspring Developmental Delay
Source: JAMA Netw Open. 2026 Jun 23;9(6):e2618804. doi: 10.1001/jamanetworkopen.2026.18804 (PMC13291893; doi:10.1001/jamanetworkopen.2026.18804)
Supplement: Supplement 2. — Data Sharing Statement [file jamanetwopen-e2618804-s002.pdf]

# Data Sharing Statement

Ohseto. Maternal Cardiovascular Health During Pregnancy and Offspring Developmental Delay. *JAMA Netw Open*. Published June 24, 2026.
doi:10.1001/jamanetworkopen.2026.18804

**Data**
**Data available:** Yes
**Data types:** Deidentified participant data
**How to access data:** The data underlying this study are held by the Tohoku Medical Megabank Organization and contain sensitive personal information. In accordance with institutional and ethical requirements, the dataset is not publicly available. De-identified data may be made available upon approval by the relevant ethics and data access committees. Please contact the corresponding author for data access enquiries.
**When available:** With publication

**Supporting Documents**
**Document types:** None

**Additional Information**
**Who can access the data:** Researchers whose proposed use of the data has been approved.
**Types of analyses:** De-identified data
**Mechanisms of data availability:** After approval of a proposal
